# Phylogenomics reveals the evolution of floral traits associated with pollinators and pollinator–prey conflict within the carnivorous *Pinguicula* subgenus *Temnoceras*

**DOI:** 10.1002/ajb2.70156

**Published:** 2026-01-31

**Authors:** Yunjia Liu, Qianshi Lin, Steven J. Fleck, Martín Mata‐Rosas, Enrique Ibarra‐Laclette, Tanya Renner

**Affiliations:** ^1^ Department of Entomology The Pennsylvania State University 501 ASI Building, University Park 16802 PA USA; ^2^ Sam and Ann Barshop Institute for Longevity and Aging Studies Department of Biochemistry and Structural Biology UT Health San Antonio, 4939 Charles Katz Drive San Antonio 78229 TX USA; ^3^ Department of Biology Lakehead University 955 Oliver Rd, Thunder Bay, ON P7B 5E1 Canada; ^4^ Department of Biological Sciences University at Buffalo Buffalo 14260 NY USA; ^5^ Red Manejo Biotecnológico de Recursos Instituto de Ecología, A.C. Carretera Antigua a Coatepec No. 351, Col. El Haya. C.P., Xalapa Veracruz 91073 Mexico; ^6^ Red de Estudios Moleculares Avanzados Instituto de Ecología A.C. Carretera Antigua a Coatepec No. 351, Col. El Haya. C.P., Xalapa Veracruz 91073 Mexico

**Keywords:** ASTRAL, carnivorous plants, evolution, *ndh* gene loss, phylogenomics, *Pinguicula*, pollination syndromes, pollinator‐prey conflict (PPC)

## Abstract

**Premise:**

The carnivorous plant genus *Pinguicula* (Lentibulariaceae) exhibits remarkable floral diversity associated with pollination, particularly in the largest subgenus *Temnoceras*, which spans Mexico and Central America. Despite this diversity, the relationships between species and the evolution of key floral traits remain unresolved. Here, we employed whole‐genome sequencing to reconstruct a robust phylogeny and examine the evolution of pollination syndromes and potential pollinator–prey conflicts.

**Methods:**

We generated nuclear and plastid genomic data for 32 *Pinguicula* species. Phylogenetic relationships were inferred using 2189 BUSCO loci analyzed through ASTRAL. Morphological traits associated with pollination and carnivory were assessed with ancestral state reconstruction, principal component analysis, and phylogenetic linear models. Loss and pseudogenization of *ndh* genes implicated in potential shifts in trophic strategies were evaluated in both nuclear and plastid genomes.

**Results:**

Our genome‐scale phylogeny resolved six monophyletic clades within *Temnoceras*, refining infrageneric classification. Most *ndh* genes are either lost or pseudogenized across both genomic compartments. Floral morphology strongly clusters by pollinator type, with fly‐pollinated species forming a distinct clade characterized by cylindrical spurs and tubes. Ancestral reconstructions indicate multiple independent transitions in spur and tube morphology. Phylogenetic linear modeling revealed a significant evolutionary correlation between scape length and carnivorous leaf area, suggesting that spatial separation may represent an adaptive response to mitigate pollinator–prey conflict.

**Conclusions:**

This study provides a refined phylogenetic framework for *Pinguicula* subgenus *Temnoceras* and highlights how pollinator specialization and carnivory‐related traits contribute to floral evolution.The repeated loss of *ndh* genes implies relaxed selective pressure on photosynthesis‐related pathways in these carnivorous species.

The Lentibulariaceae is a family of three carnivorous genera: *Pinguicula*, *Genlisea*, and *Utricularia*. *Pinguicula* L., commonly known as the butterworts, includes over 111 described species, with the number steadily increasing due to ongoing discoveries (Casper, 1966; Juárez‐Gutiérrez et al., 2018; Shimai et al., 2021; Zamudio et al., 2023, López‐Pérez et al., 2024, 2025). Although members of this genus share a basic morphological blueprint, they vary remarkably in life form, life cycle, and the shape, size, and coloration of both leaves and flowers. *Pinguicula* species produce a basal rosette of carnivorous leaves and a shallow root system, from which one or more scapes arise, bearing either hermaphroditic or zygomorphic flowers characterized by a tubular corolla and a nectar spur. Each flower has five sepals and displays a bilabiate structure, with two sepals forming the upper lip and three forming the lower lip. Along with the corolla shape, color, and pattern, the length and morphology of the floral tube and nectar spur also vary extensively among species (Barnhart, 1916; Casper, 1966; Fleischmann and Roccia, 2018; Fleischmann, 2021; Shimai et al., 2021). These floral traits were historically used to delineate species and establish taxonomic groupings within the genus. Originally, *Pinguicula* was divided into three subgenera by Casper (1966): *Temnoceras* Barnhart, *Isoloba* Barnhart, and *Pinguicula*. Subgenus *Isoloba* includes species with isolobate corollas, typically exhibiting five sub‐actinomorphically arranged lobes of nearly equal shape and size. In contrast, subgenus *Pinguicula* comprises species with strongly bilabiate, dark‐colored corollas, characterized by two smaller upper lobes and three larger lower lobes, including a prominently expanded median lobe on the lower lip. Subgenus *Temnoceras* shares a similar corolla structure with *Pinguicula*, but its flowers tend to have paler coloration (Casper, 1966; Fleischmann, 2021).

As molecular technologies have advanced, traditional morphology‐based classifications have been reevaluated through phylogenetic reconstructions using molecular markers (Fleischmann and Roccia, 2018; Shimai et al., 2021). The infrageneric classification of *Pinguicula* was revised with the incorporation of markers such as *matK* and ITS (Kondo and Shimai, 2006; Shimai and Kondo, 2007; Fleischmann and Roccia, 2018). Both the ITS and plastid trees assigned the three subgenera recognized by Casper (1966): subgenus *Isoloba*, *Pinguicula*, and *Temnoceras*. The internal sectional structure was subsequently reassigned as follows: subgenus *Isoloba* includes sections *Isoloba* Casper, *Cardiophyllum* Casper, *Pumiliformis* (Casper) Roccia & A. Fleischmann, and *Ampullipalatum* Casper; subgenus *Pinguicula* retains section *Pinguicula*; and subgenus *Temnoceras* encompasses sections *Temnoceras* Casper, *Micranthus* Casper, *Nana* Casper, and *Heterophylliformis* (Casper) A. Fleischmann & Roccia.

According to Shimai et al. (2021), species relationships within the subgenera of *Pinguicula* exhibit a clear pattern of association with geographic distribution. As noted by Fleischmann and Roccia (2018), subgenus *Temnoceras*, section *Temnoceras*, includes species from Central America (42 spp.), Cuba (9 spp.), and Hispaniola (1 sp.), while section *Heterophylliformis* contains the single South American species *P. elongata* (Beck et al., 2008). In contrast, the section *Micranthus* of *Temnoceras* includes *P. alpina*, native to Europe and northeastern Asia (Shimai et al., 2021), and section *Nana* comprises four species distributed across the Northern Andes, alpine regions, and (sub)arctic Eurasia (Fleischmann and Roccia, 2018). Within subgenus *Pinguicula*, section *Pinguicula* encompasses species from temperate Eurasia and the Mediterranean (23 spp.), as well as two species from temperate North America. Subgenus *Isoloba* exhibits the widest geographical range, with species occurring in the Northern and Central Andes (3 spp.), the South Andean foothills and Patagonia (2 spp.), the coastal southeastern United States (6 spp.), the Mediterranean and Asia (6 spp.), and Atlantic Europe (1 sp.). The phylogeographic structure of the genus was further analyzed and categorized into nine biogeographic clades by Shimai et al. (2021). Section *Temnoceras* sensu Fleischmann and Roccia (2018), which includes all Central American and Caribbean species of *Pinguicula*, has consistently formed a monophyletic group in multiple phylogenetic analyses (Fleischmann and Roccia, 2018; Shimai et al., 2021). Shimai et al. (2021) further resolved this section into four well‐supported monophyletic lineages with shared morphological or geographical features. These were later formalized by Fleischmann (2021) as four subsections: (1) subsection *Temnoceras* Barnhart (1916), comprising all annual Mexican species; (2) subsection *Orcheosanthus* DC. (1844), which includes species with succulent carnivorous leaves with margins involute only near the tip or not at all, found primarily in the Sierra Madre Occidental through Central America and the Sierra Madre del Sur; (3) subsection *Agnata* Casper (1963), emend. A. Fleischmann, including species with larger, thinner leaves with fully involute margins, mostly from the Sierra Madre Oriental; and (4) subsection *Homophyllum* Casper (1963), encompassing all Caribbean species. These lineages correspond to Clades VI through IX of Shimai et al. (2021). Despite these advances, the current taxonomic framework of *Pinguicula* continues to face limitations, particularly in terms of low phylogenetic resolution and inconsistencies among different data sets.

Overall, phylogenetic relationships among *Pinguicula* species have shown inconsistencies with traditional morphology‐based classifications. Although Casper's early taxonomic groupings have been reevaluated through molecular phylogenetics, floral variation among closely related species remains a topic of significant interest. It is widely recognized that diverse floral phenotypes often reflect specialization to particular pollinators and that selection by pollinators can drive floral diversification (Kölreuter, 1761; Fenster et al., 2004). Floral traits whose directional evolution is shaped by pollinator‐mediated selection are referred to as “pollination syndromes” (Delpino, 1874; Fenster et al., 2004; Dellinger, 2020). Within *Pinguicula*, a wide array of floral traits, such as corolla shape and color, nectar rewards (e.g., nectar and edible starch trichomes), and the shape and length of the nectar spur, which vary markedly among species and are likely associated with the diverse pollination strategies of the genus (Whittall and Hodges, 2007; van der Kooi et al., 2019; Vandelook et al., 2019; Lustofin et al., 2020). Species with long nectar spurs, funnel‐shaped floral tubes, and dark‐colored flowers (e.g., bright red, pink, or purple) are considered adaptations to long‐tongued pollinators such as butterflies and hummingbirds (Fleischmann, 2021). This pollinator‐driven adaptation of a prolonged and rewarded spur has been documented in other angiosperm lineages, such as *Diascia* spp., *Aquilegia* spp., and *Habenaria* orchids, where spur length evolution closely matches the morphology of specialized pollinators (Whittall and Hodges, 2007; Sletvold et al., 2010, Hollens‐Kuhr et al., 2021). Certain species confirmed to be butterfly‐pollinated (psychophilous), like *P. moranensis*, provide nectar in the spur as a reward (Zamudio, 2001; Lustofin et al., 2020). In contrast, species pollinated by flies (myophily) or bees (melittophily) often have edible nonglandular trichomes within the floral tube and at the base of the spur, which serve as rewards (Lustofin et al., 2020, 2023). Detailed anatomical studies have shown that these trichomes can be glandular or nonglandular, and their position and structure vary across species, potentially reflecting adaptations to specific pollinators (Lustofin et al., 2020; 2023). However, observational data sometimes contradict expected pollination syndromes. For instance, while the floral morphology of *P. moranensis* aligns with psychophily, this species has also been observed to attract bee visitors (Pérez‐Alva et al., 2022). Nevertheless, floral traits in *Pinguicula* generally correlate with particular pollinator groups, and species‐sharing pollination strategies tend to form well‐supported monophyletic clades (Shimai et al., 2021; Lustofin et al., 2023).

The distinctive flowers of *Pinguicula* species underscore the complex plant–insect interactions between these carnivorous plants and their pollinators. Beyond pollination, *Pinguicula* also engages in predator–prey interactions, relying on insects as a source of essential nutrients. This dual role introduces an ecological conflict: Both pollinators and prey may be attracted to and captured by the same plant, creating a trade‐off between reproductive success and nutrient acquisition. This dilemma was first described by Juniper et al. (1989) as the “prey/pollinator paradox” and later formalized by Zamora et al. (1999) as the “pollinator–prey conflict”. To address this paradox, Jürgens et al. (2012) proposed an optimality model framing pollinator–prey conflict as a trade‐off between the benefits of prey capture and successful pollination. Their model highlighted two principal mechanisms by which carnivorous plants may mitigate this conflict. The first involves a spatial or temporal separation between flowers and trapping structures. *Pinguicula* exemplifies this strategy by producing long scapes that elevate flowers away from the carnivorous leaf rosette, thereby reducing the likelihood of pollinator entrapment (Ellison and Gotelli, 2001; Fleischmann and Roccia, 2018; Tagawa, 2020). The second mechanism entails the use of distinct visual and/or chemical cues by flowers and traps to attract different insect groups (Schaefer and Ruxton, 2008; Jürgens et al., 2012; El‐Sayed et al., 2016; Ojeda et al., 2021). More recently, the efficacy and ecological prevalence of these strategies in *Pinguicula* and other carnivorous plant lineages have been reassessed using broader comparative perspectives and field‐based syntheses (Luna‐Samano et al., 2024). These studies, together with targeted assays, indicate that multimodal divergence, combining spatial separation with differences in chromatic and volatile cues, may represent an important mechanism for minimizing pollinator–prey overlap, particularly under ecological scenarios with high insect visitation rates (Jürgens et al., 2012; El‐Sayed et al., 2016; Cuevas et al., 2023).

Taxonomic classifications within *Pinguicula* have been refined through the use of molecular markers, which have enhanced our understanding of the distribution, speciation, carnivory, and pollinator‐driven evolutionary patterns of the genus. Nonetheless, unresolved questions persist, particularly regarding species relationships due to incongruences among phylogenetic trees, the evolution of floral characters, and potential evolutionary trends related to pollinator‐prey conflicts in *Pinguicula*.

In this study, we focused on *Temnoceras*, the largest subgenus within *Pinguicula*, whose species are primarily distributed across Mexico and Central America. We employed a whole‐genome sequencing approach to achieve greater phylogenetic resolution within the subgenus and to investigate the evolution of morphological traits. Specifically, we (1) refined and updated the taxonomic classification of *Pinguicula* based on phylogenetic reconstructions from nuclear and chloroplast loci obtained through whole‐genome sequencing of 32 species, (2) analyzed *ndh* gene loss and pseudogenization in both nuclear and plastid genomes to further clarify species relationships, (3) explored the evolutionary history of floral traits associated with pollination, and (4) evaluated whether morphological character evolution reflects phylogenetic signal, is shaped by pollinator‐driven selection, or results from adaptations to pollinator–prey conflict.

## MATERIALS AND METHODS

### Species identification and sampling

This study focused on *Pinguicula* species traditionally assigned to the subgenus *Temnoceras* (Appendix S1: Table S1). All living plants utilized were either sourced directly from verified collections at the Instituto de Ecología, A.C. (Xalapa, Veracruz, Mexico) or purchased from California Carnivores (Sebastopol, CA, USA). Species identifications were verified through morphological comparisons with published descriptions and/or type material references. Plants were maintained in either tissue culture or within soil, then leaves or whole plants were flash‐frozen and stored at –80°C until DNA extraction. Voucher specimens were prepared for all species and deposited into The Pennsylvania Agricultural College (PAC) Herbarium (University Park, PA, USA).

### DNA extraction and whole‐genome sequencing

DNA was extracted using the Qiagen DNeasy Plant Pro Kit (Qiagen, Germantown, MD, USA). Briefly, 300 mg of frozen plant tissue was used per extraction. To optimize the protocol, the sample mass was increased to 300 mg and the lysis buffer volume to 500 μL. Tissue was homogenized using a FastPrep‐24 (MP Biomedicals, Irvine, CA, USA) with the following settings: 5.5 m/s for 50 s, repeated twice. For samples yielding relatively low amounts of DNA, an additional extraction was performed, combined with the initial product, and diluted with 100 μL of nuclease‐free water. DNA quantity and quality were assessed using a Nanodrop 2000c and a Qubit 1.0 fluorometer (Thermo Fisher Scientific, Waltham, MA, USA). Samples (≥200 ng per sample, ≥5 ng/μL) were submitted to Novogene (Sacramento, CA, USA) for quality control, library preparation, and 2 × 150 bp Illumina NovaSeq whole‐genome sequencing.

### Genome assembly and ploidy estimation

Illumina reads were trimmed using Trimmomatic v0.39 (Bolger et al., 2014) to remove adapter sequences (ILLUMINACLIP: TruSeq3‐PE.fa:2:30:10) and filtered for quality with the parameters –MINLEN:36 and –SLIDINGWINDOW:4:15. All species were assembled de novo using MaSuRCA v4.1.0 (Zimin et al., 2013). Assemblies were refined using Purge Haplotigs (Roach et al., 2018) to reduce heterozygosity, and both pre‐ and post‐purging assemblies were evaluated with QUAST v5.2.0 (Gurevich et al., 2013). Assembly statistics were generated with the Quality Assessment Tool for Genome Assemblies (QUAST) v5.2.0 (Gurevich et al., 2013), and completeness was assessed using Benchmarking Universal Single‐Copy Orthologs (BUSCO v5.5.0 with the eudicots_odb10 data set (Manni et al., 2021). For *Pinguicula* species lacking recorded ploidy levels, we used Smudgeplot v0.2.2 (Ranallo‐Benavidez et al., 2020) to estimate ploidy and visualized the results using GenomeScope (Ranallo‐Benavidez et al., 2020). The resulting smudgeplots are available in Appendix S2.

### Chloroplast genome assembly and annotation

Plastomes were assembled from trimmed paired‐end reads of *Pinguicula* using GetOrganelle (Jin et al., 2020) with default settings: ‐F embplant_pt to specify the plastome as the target organelle genome type and ‐R 10 for a maximum of 10 extension rounds. Five *k*‐mer sizes were tested to optimize assembly. For *P. caerulea* and *P. kondoi*, the extension parameter was increased to ‐R 25 to enable recovery of a complete circular plastome. Circular plastomes were visualized using Bandage (Wick et al., 2015), and annotations were generated via the GeSeq Chlorobox online platform (Tillich et al., 2017).

### Phylogenetic reconstructions

#### BUSCO‐based phylogenetic reconstructions

BUSCO version 5.4.4 (Manni et al., 2021) was used to reconstruct two distinct phylogenies. BUSCOs were extracted from *Pinguicula* MaSuRCA post‐purged haploid genome assemblies using the eudicots_odb10 data set (Manni et al., 2021). Single‐copy BUSCO protein sequences from each assembly were retrieved and grouped using an in‐house script (Appendix S3). Each protein FASTA file was aligned with MAFFT v7.471 (Nakamura et al., 2018) using default parameters and trimmed with trimAl v1.4.rev22 (Capella‐Gutiérrez et al., 2009) under the automated1 option. For each aligned and trimmed BUSCO data set containing four or more species, phylogenetic trees were reconstructed using IQ‐TREE v2.1.4 (Nguyen et al., 2015), with 1000 SH‐like approximate likelihood ratio test (SH‐aLRT) replicates, 1000 ultrafast bootstrap replicates, and model selection was performed with ModelFinder (Nguyen et al., 2015). The resulting gene trees were used as input for species tree inference in ASTRAL 5.7.8 (Zhang et al., 2018) with default settings. As ASTRAL estimates branch lengths for internal nodes only, terminal branch lengths were annotated by calculating substitution‐per‐site units (SUs) derived from the gene trees. BUSCO gene tree branch lengths were used under an extension of the multispecies coalescent (MSC) model to estimate rates across the species tree using CASTLES (Tabatabaee et al., 2023). Alignments, IQ‐TREE gene trees, and the ASTRAL consensus tree are available in Appendix S3.

#### Chloroplast phylogenetic reconstruction

We retrieved 80 plastid protein‐coding genes (Appendix S1: Table S2) using the blastn program from BLAST + NCBI (version NCBI‐BLAST‐2.2.30 + ; Camacho et al., 2009), with *Pinguicula alpina* (NC056190) and *Utricularia tenuicaulis* (NC058517) as query sequences (Appendix S1: Table S2). Each gene was aligned using MUSCLE (Edgar, 2004), then manually adjusted in Mesquite v3.81 (Maddison and Maddison, 2023) (Appendix S4).

The plastome phylogenetic tree was reconstructed using IQ‐TREE v2.1.4 with 1000 SH‐like approximate likelihood ratio test (SH‐aLRT) replicates and 1000 ultrafast bootstrap replicates; the best‐fitting model for each alignment was selected using ModelFinder. This approach also allowed us to recover chloroplast *ndh* genes, which were included in our analysis of *ndh* gene loss across *Pinguicula* and other Lentibulariaceae members. The resulting plastome phylogeny is available in Appendix S4.

### Nuclear *ndh* gene identification

Amino acid sequences encoded by nuclear *ndh* genes from *Arabidopsis thaliana* (*ndhM*, *ndhU*, *ndhS*, *ndhT*, *ndhO*, *ndhN*, and *ndhL*) were downloaded from NCBI (Appendix S1: Table S2). These sequences were then used as reference queries in tblastn searches against the *Pinguicula* genome and plastome assemblies, using an e‐value cutoff of 1e–5 (NCBI‐BLAST v2.11; Camacho et al., 2009) to identify candidate genes. The absence of hits was interpreted as gene loss from the nuclear and plastid genomes. The completeness of each hit was evaluated based on query coverage, and gene functionality was assessed by manually screening alignments for premature stop codons indicative of pseudogenization. The same *A. thaliana* NDH amino acid sequences were also used as queries in tblastn searches against *Genlisea aurea*, *Salvia splendens*, and *Utricularia gibba* using the same e‐value threshold (≤1e–5).

### Flower characters survey and pollinator data collection

**Figure 1:**
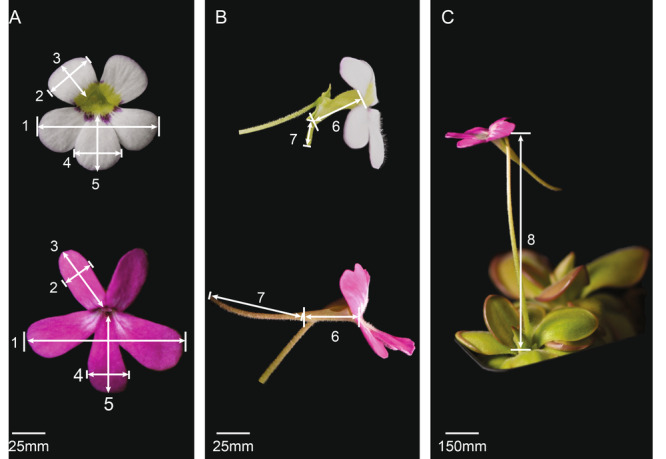
Survey standard for measuring *Pinguicula* flower quantitative characters of (A) subisolobate (upper) and bilabiate flower across (1), upper corolla width (2) and length (3), lower corolla width (4) and length (5); (B) flower tube length (6) and flower spur length (7); (C) scape length (8). Additional quantitative traits (scape number and flower length including the flower spur) and qualitative traits were studied and are detailed in Appendix S1: Table S3.

Morphological characters were comprehensively compiled from published sources (Lampard et al., 2016; Shimai, 2017). The resulting phylogenetic reconstruction served as a framework for examining infrageneric classification and the evolution of floral traits in *Pinguicula*. A wide range of qualitative and quantitative floral traits (categorized under traditional pollination syndromes; Figure 1) and leaf traits and pollinator records were gathered from the literature and direct observations of plant material (Lampard et al., 2016; Shimai, 2017). These traits included flower size, corolla color, corolla length and width, spur length, tube length and width, and scape length; summer leaf length and width, leaf shape and margin; winter leaf length and width, leaf shape and margin; and life form (Figure 1; Appendix S1: Table S3). In addition to the direct measurements, we included four derived traits for phylogenetically independent contrast analyses: (1) leaf number, (2) leaf length and width while flowering (summer or winter leaf), (3) leaf area (length × width of the flowering leaf), and (4) total carnivorous leaf area (length × width × leaf number while flowering).

### Comparative phylogenomic analyses of character states

Phylogenetic relationships for *Pinguicula* subgenus *Temnoceras*, with the functional outgroup species *P. caerulea* from subgenus *Isoloba*, were reconstructed using the ASTRAL species tree based on BUSCO genes. *Pinguicula caerulea* was designated as the functional outgroup and used to root the phylogeny. Qualitative and quantitative floral and leaf traits were analyzed and visualized using the PCAmix R package, with a 95% confidence ellipse overlaid according to pollinator type (Chavent et al., 2014).

Scape length in carnivorous plants has been considered a potential factor influencing pollinator‐prey conflicts (Anderson, 2010; Youngsteadt et al., 2018). To assess whether scape length is evolutionarily correlated with carnivorous leaf area, we conducted phylogenetic linear model (PLM) tests using the phylolm package in R (Tung Ho and Ané, 2014). We first examined the relationship between scape length and two leaf‐related morphological traits: total carnivorous leaf area and leaf length at flowering (used as a proxy for plant size), separately and in combination. Leaf measurements were taken from the rosette during flowering (Lampard et al., 2016; Shimai, 2017), whether during the summer or winter phase, depending on *Pinguicula* species phenology (Appendix S1: Table S3). All variables were log‐transformed.

Model 1: log(scape length) ~ log(carnivorous leaf area)

Model 2: log(scape length) ~ log(leaf length)

Model 3: log(scape length) ~ log(carnivorous leaf area) + log(leaf length)

Allometric correction model: residuals (Model 2) ~ log(carnivorous leaf area)

Each model was fitted to the phylogeny under Pagel's lambda (Pagel, 1999) to test for phylogenetic signals in the residuals. Model performance was compared using the Akaike information criterion (AIC). To evaluate whether the association between scape length and carnivorous leaf area was independent of allometric scaling with plant size, we extracted residuals from Model 2 and used them as a variable in a regression model with leaf area.

Among floral traits, flower tube shape, spur type, and spur length have been studied as key features associated with pollination syndromes (Whittall and Hodges, 2007; Anderson et al., 2014). Ancestral state reconstructions for continuous traits in *Pinguicula* were estimated using the fastAnc function in the phytools R package, based on a Brownian motion model with phenotypic values estimated at internal nodes and 95% CIs. Marginal ancestral states for qualitative floral traits, including flower tube and spur types, were reconstructed using the link.anc function in phytools (Revell, 2012). The phylogenetic signal in log‐transformed values for flower tube and spur length was assessed using Blomberg's *K* (Blomberg et al., 2003) and Pagel's lambda (Pagel, 1999) via the phylosig function. To explore patterns and rates of transitions among different flower tube and spur types, we applied the fitDiscrete function to compare model fits for equal rates (ER), symmetric (SYM), and all rates different (ARD) models. Model selection was based on pairwise likelihood ratio tests using the lrtest function in phytools. The phylogenetic reconstruction, morphological collection input, full code and resulting outputs for principal component analysis (PCA), phylogenetic linear models, and ancestral state reconstruction tests are available in Appendix S5.

## RESULTS

### Genome assemblies and ploidy estimation

We sequenced 32 species from the largest section of *Pinguicula*, *Temnoceras*. For each species, approximately 29.9–53.1 Gb of raw sequencing data were obtained, with an average of 36 Gb and over 60× coverage (Appendix S1: Table S4). Genome assembly quality was assessed using N50 contig length and BUSCO completeness scores (Appendix S1: Table S5). Following PurgeHaplotigs processing, N50 values increased by an average of 7% across species. In the purged assemblies, GC content ranged from 38% to 39.52%, and N50 values ranged from 10,242 to 29,390. On average, more than 80% of BUSCOs were recovered as complete, indicating high‐quality assemblies suitable for downstream analyses (Appendix S1: Table S5). SmudgePlot analyses suggested that all *Pinguicula* species in the data set are diploid, though chromosome number and haplotype composition varied among species (Appendix S2; Appendix S1: Table S6).

We successfully assembled complete, circularized plastomes for 29 *Pinguicula* species, excluding *P. macrophylla*, *P. laxifolia*, and *P. zamudioana*. The assembled plastomes had an average length of approximately 146 kb.

### Phylogenomic reconstructions

**Figure 2:**
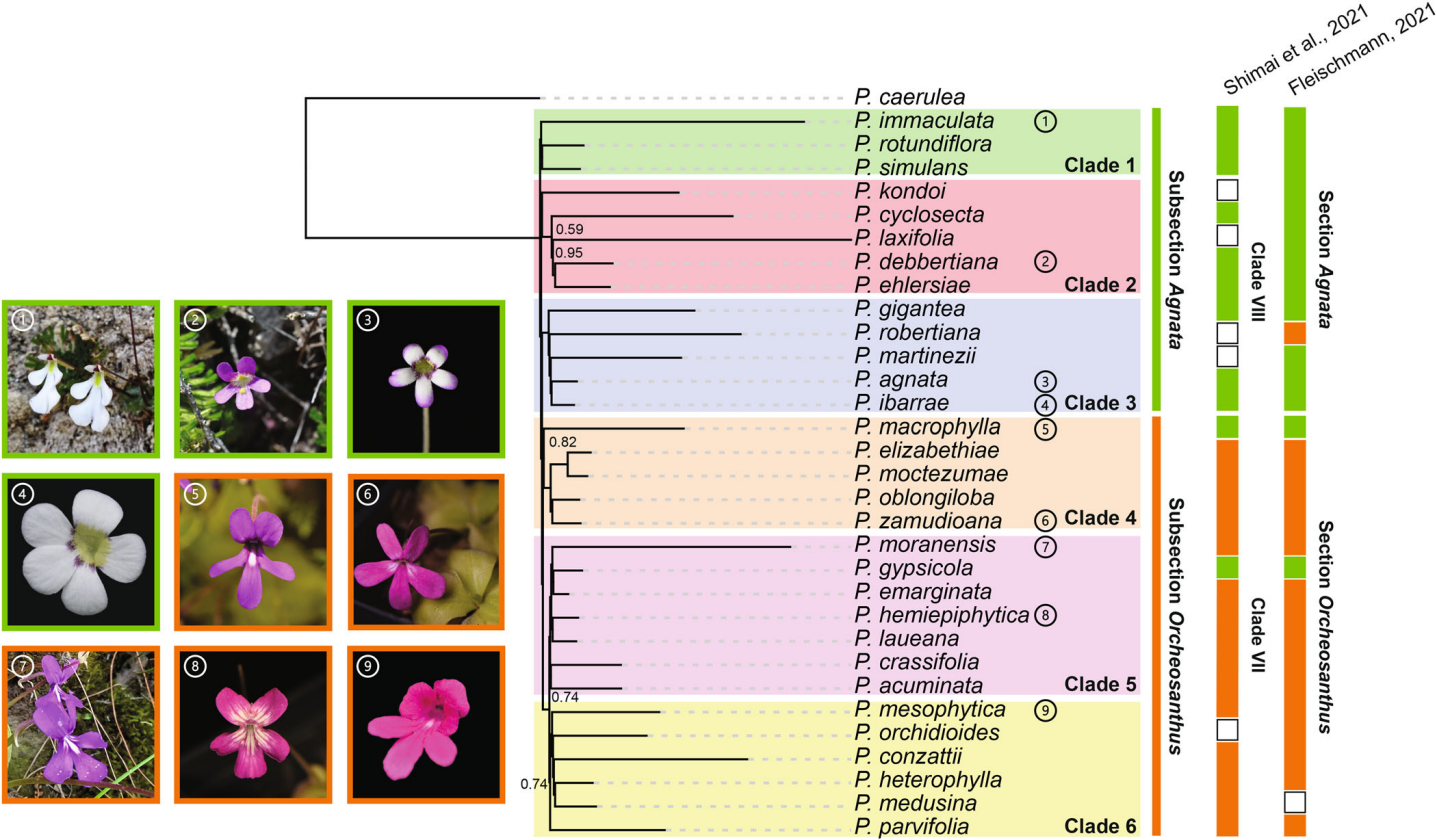
Summary of classification of *Pinguicula* subgenus *Temnoceras* section *Agnata* and section *Orcheosanthus* ASTRAL tree based on BUSCO genes. Number within circle = photograph number. Species tree branch lengths were annotated by calculating substitution‐per‐site units (SU) derived from the gene trees. Branch support values were local posterior probabilities calculated by ASTRAL. Clades 1–6 are based on the phylogenetic reconstruction presented in this study. Shimai et al. (2021) and Fleischmann (2021) classification reported on the right. *Pinguicula* photographs taken by Martín Mata‐Rosas (1, 2, 7) and Yunjia Liu (3, 4, 6, 8, 9).

Based on the phylogenetic relationships inferred from the ASTRAL species tree, the 32 *Pinguicula* species analyzed in this study were assigned to six monophyletic groups (clades) (Figure 2). This sampling fills gaps in subgenus *Temnoceras* coverage compared to that of Shimai et al. (2021); species not included by Shimai et al. (2021) and Fleischmann (2021) are indicated with open squares in Figure 2. Following the ITS phylogeny of Shimai et al. (2021) and the classification proposed by Fleischmann (2021), we assigned *Pinguicula* species with nuclear genome data in Clades 1–3 to (sub)section *Agnata* and species in Clades 4–6 to (sub)section *Orcheosanthus*. The ASTRAL local posterior probability (localPP) for the split between (sub)sections *Agnata* and *Orcheosanthus* was 0.99, indicating strong support for this sectional distinction. All backbone nodes received support values above 0.99, and most internal nodes were also highly supported. Across the phylogeny, several species exhibit unusually long branch lengths (e.g., *P. immaculata*, *P. laxifolia*, *P. moranensis*), potentially reflecting lineage‐specific increases in genome‐wide loci substitution rates. Notably, *Pinguicula gypsicola* was reassigned to (sub)section *Orcheosanthus* with strong support, contrasting with its previous placement in (sub)section *Agnata* by Shimai et al. (2021) and Fleischmann (2021).

**Figure 3:**
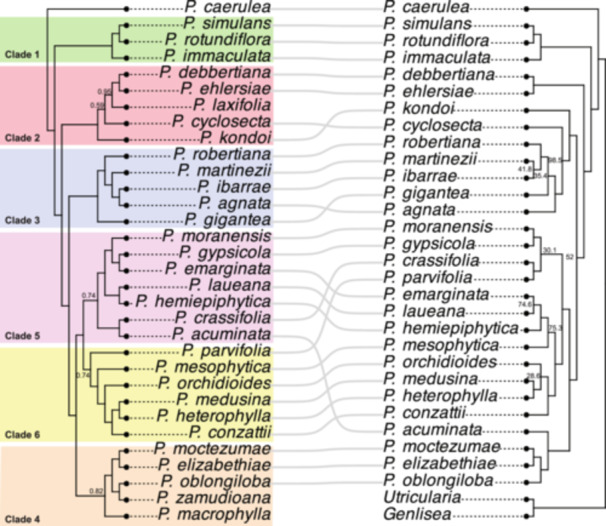
Comparison of the *Pinguicula* ASTRAL species tree based on BUSCO genes and species tree based on plastid genes. The figure shows topological congruence between the species tree generated by ASTRAL‐III based on 2198 single copy BUSCOs gene alignments tree and plastome tree generated by 80 plastid protein coding genes. The ASTRAL and plastome tree support values (local posterior probabilities and Ultrafast bootstrap) are labeled on the nodes, but those of greater than 0.99 and 99 are collapsed.

The plastid genome phylogeny of *Pinguicula* showed strong support along the backbone, with the exception of the clade containing *P. ehlersiae* and *P. debbertiana*, which received relatively weak support in both ultrafast bootstraps (UFBoot = 52) and SH‐aLRT tests (SH‐aLRT = 67). Several internal nodes also exhibited low bootstrap values, including the grouping of *P. martinezii* and *P. ibarrae* (UFBoot = 41.8), *P. gigantea* (UFBoot = 35.4), *P. medusina*, *P. heterophylla*, and *P. orchidioides* (UFBoot = 28.6), as well as *P. moranensis*, *P. gypsicola*, *P. crassifolia*, and *P. parvifolia* (UFBoot = 30.1). Only one strongly supported monophyletic cluster was recovered for *P. martinezii* and six other species (Figure 3).

### Loss of photosynthesis‐related genes within the chloroplast and nuclear genomes of *Pinguicula*

Upon examining the genome assemblies for missing BUSCO genes, we identified 88 BUSCOs absent across all *Pinguicula* species, including genes such as NAD(P)H‐quinone oxidoreductase subunit N (BUSCO gene code 135370at71240) and chloroplastic NAD(P)H‐quinone oxidoreductase subunit U (BUSCO gene code 138217at71240; Appendix S1: Table S7). Moreover, taxonomic relationships in the plastid phylogeny reflect consistent patterns of gene retention and loss among taxa with respect to photosynthesis‐related gene complexes. For instance, *P. immaculata*, *P. rotundiflora*, and *P. simulans* retain *ndhB* and *ndhE* (complex I), *ndhL* (subcomplex A), and *PnsL5* (lumenal subcomplex), but have lost genes from subcomplex B (*PnsB1*, *PnsB2*), the lumenal subcomplex (*PnsL1*), and linker components (*Lhca5*).

**Figure 4:**
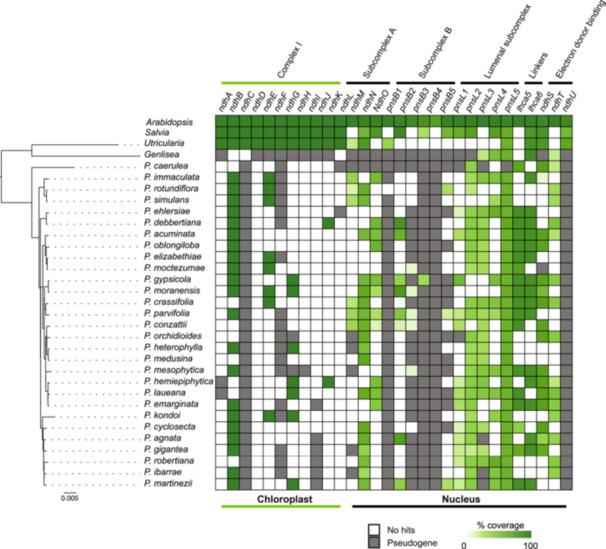
*Pinguicula* plastome phylogeny and *ndh* gene loss. The plastome phylogeny of *Pinguicula* was reconstructed based on 80 plastid genes using the maximum likelihood method. Branch support values (Ultrafast bootstrap) below 99 are indicated. The accompanying heatmap shows the completeness of *ndh* genes in both plastome and genomes, with color intensity corresponding to the sequence coverage for each gene.

Similarly, the clade including *P. kondoi*, *P. cyclosecta*, *P. agnata*, *P. gigantea*, *P. robertiana*, *P. ibarrae*, and *P. martinezii* (UFBoot = 100; Figure 4) displays similar patterns of gene retention (e.g., *ndhB*, *ndhM*, *PnsL5*) and loss (e.g., *ndhI* and subcomplex B genes). Interestingly, the weak support for the sister relationship between *P. ibarrae* and *P. martinezii* (UFBoot = 41.8; Figure 4) may correspond with comparable plastid gene losses shared by these species.

Compared to non‐carnivorous controls (*Arabidopsis* and *Salvia*), all *Pinguicula* species lack the *ndhC* gene. Most other plastid *ndh* genes are either lost or pseudogenized across *Pinguicula*, except *ndhB*, which persists as a pseudogene in six species. In the nuclear genome, genes associated with subcomplexes A and B and electron donor binding (*ndhO*, *ndhU*, *PnsB3*, *PnsB4*) are either completely absent or pseudogenized, while genes encoding lumenal subcomplex and linker components show variable patterns of loss or pseudogenization (Figure 4).

### Pollinator and prey conflict in *Pinguicula*

**Figure 5:**
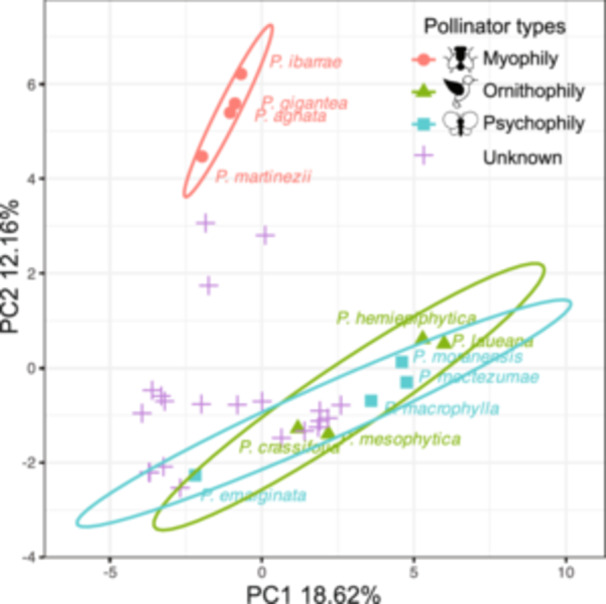
PCA plot of *Pinguicula* species variation based on flower quantitative and qualitative characters. The plot was generated using the R package mixPCA, and 95% cover rate ellipse were added based on the pollinator types (red circle: myophily; green triangle: ornithophily; blue square: psychophily; purple cross: unknown record of pollinator). Input data collection includes 10 quantitative and four qualitative floral traits, six quantitative and four leaf characters, and three plant characters.

To examine the relationship between morphological traits and pollinator types in *Pinguicula*, we performed a principal component analysis (PCA) incorporating both quantitative and qualitative characters, including features associated with pollination syndromes. The first two principal components effectively separated species into distinct clusters, which showed significant correspondence with pollinator categories, as visualized by 95% confidence ellipses (Figure 5). The first principal component accounted for 18.62% of the variation among species, while the second explained 12.16%. Species pollinated by birds (ornithophily) and butterflies (psychophily) formed distributions that did not overlap with those pollinated by flies (myophily). However, ornithophilous *Pinguicula* overlapped considerably with psychophilous species, suggesting shared floral traits possibly associated with long‐tongued pollinators—such as extended floral spurs and funnel‐shaped corolla tubes.

**Figure 6:**
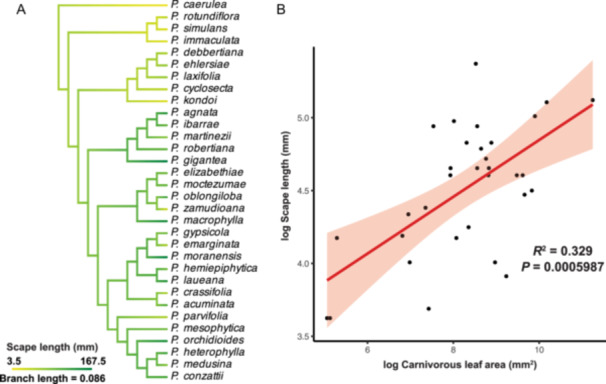
*Pinguicula* potential pollinator and prey conflict evolution. (A) Ancestral state reconstruction of *Pinguicula* scape length. (B) Phylogenetic linear model revealed that there is a significantly (*R* ^2^ = 0.329, *P* = 0.0005987) coordinated evolutionary relationship between *Pinguicula* scape length and *Pinguicula* carnivorous leaf total area while flowering.

We also used the phylogeny to infer the evolutionary history of pollination syndromes in *Pinguicula*. To identify transitions in these syndromes, we mapped scape length onto the ASTRAL species tree using maximum‐likelihood ancestral state reconstruction under a Brownian motion model (Figure 6A). The results suggest that the ancestral state was characterized by short scapes, with longer scapes having evolved independently multiple times within *Pinguicula* subgenus *Temnoceras*, notably in *P. gigantea* and *P. moranensis*.

In the phylogenetic linear model (PLM) analyses, univariate Model 1, log(scape length) ~ log(carnivorous leaf area), revealed a significant positive association and phylogenetic signal (*P* = 0.0006, adjusted *R*² = 0.31, Pagel's *λ* = 0.43), indicating that species with larger traps tend to evolve longer scapes. Similarly, Model 2, log(scape length) ~ log(leaf length), also showed a significant positive relationship (*P* = 0.0018, adjusted *R*² = 0.26, Pagel's *λ* = 0.45), suggesting that scape length scales with overall plant size (Appendix S1: Table S8).

To assess whether the association with leaf area was merely an effect of allometric scaling, we constructed a multivariate model (Model 3): log(scape length) ~ log(carnivorous leaf area) + log(leaf length). In this model, the effect of carnivorous leaf area was marginally significant (*P* = 0.05722), while the effect of leaf length was not (*P* = 0.19318), suggesting that trap size may influence scape length beyond what would be expected from body size alone.

To further disentangle the influence of plant size, we extracted residuals from Model 2 and tested their association with carnivorous leaf area. Although this test yielded a non‐significant result (*P* = 0.1526, *R*² = 0.07), the positive trend suggests a persistent tendency for species with larger traps to develop longer scapes, independent of overall size.

### Ancestral state reconstructions and phylogenetic signal test for *Pinguicula* flower characters

**Figure 7:**
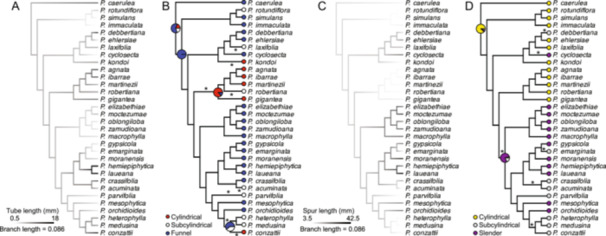
(A, B) Ancestral state reconstruction of flower tube and (C, D) flower spur in *Pinguicula*. (A) *Pinguicula* flower tube length ancestral state reconstruction. (B) *Pinguicula* flower tube shape type ancestral state reconstruction (equal rates model). Under the ER model of ancestral state reconstruction, eight transitions among flower tube types were inferred across the phylogeny. (C) *Pinguicula* flower spur length ancestral state reconstruction. (D) *Pinguicula* flower spur shape type ancestral state reconstruction (symmetric model). Under the SYM model of ancestral state reconstruction, six transitions were inferred across the phylogeny.

**Figure 8:**
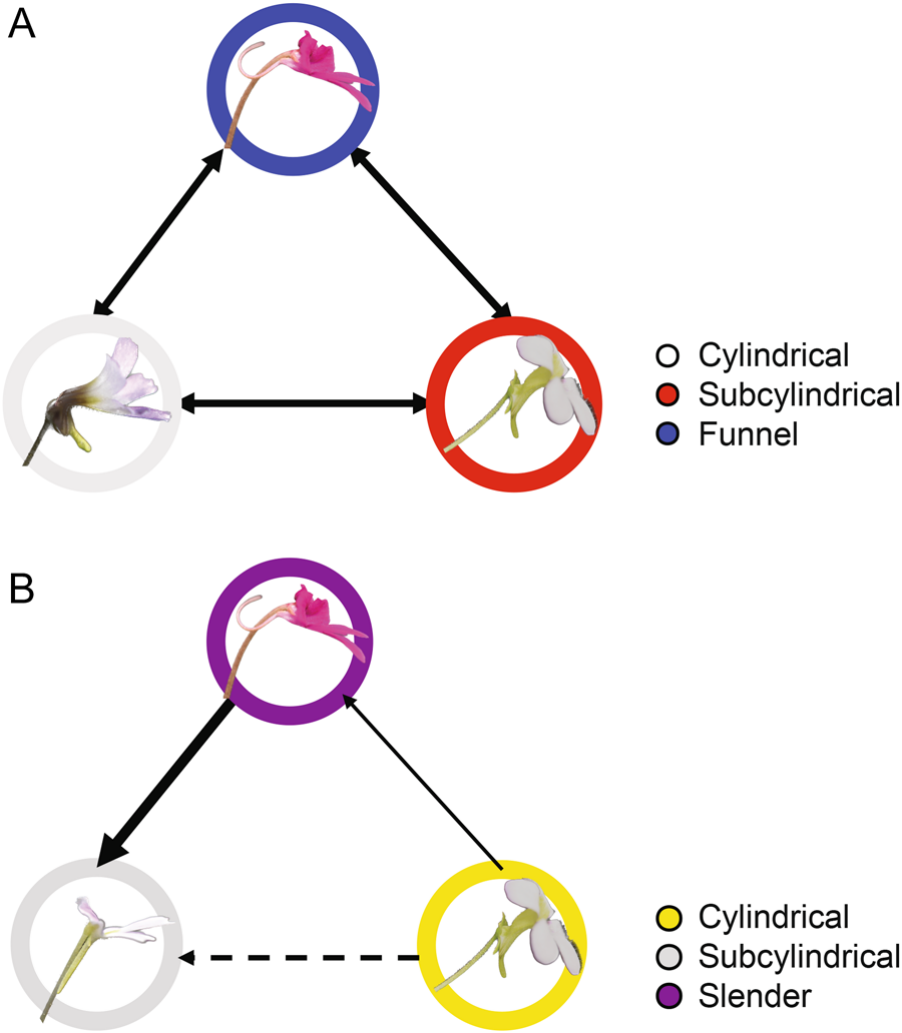
Evolutionary transitions of flower tube and spur shapes in *Pinguicula*. (A) Flower tube transition fitted to the equal rates (ER) model. (B) Flower spur transition fitted to the symmetric model (SYM) model. Arrows indicate inferred transitions among floral types. Arrow thickness is proportional to the transition rate, with dashed arrows denoting the lowest estimated rate. Detailed transition rates derived from the *q*‐matrix are provided in Appendix S1 (Table S10).

To investigate pollination syndromes associated with the flower tube and spur, we performed character transition analyses for both flower tube and spur length, as well as for their shape states (Figure 7; Appendix S1: Table S9). Flower tube length in *Pinguicula* was reconstructed across the phylogeny (Figure 7A), and the results suggest that this trait may exhibit a phylogenetic signal. Ancestral state reconstructions further indicated at least eight independent transitions in flower tube shape (Figure 7C). Species with longer branch lengths also tend to exhibit divergent morphologies. For example, *P. robertiana* has a subcylindrical tube, while related species in the same clade typically have cylindrical ones; conversely, *P. conzattii* displays a cylindrical tube in contrast to the subcylindrical condition of closely related taxa (Figure 2). To assess transition rates, we tested phylogenetic signal models and found that the equal rates (ER) model best fit the data, suggesting that transitions among tube shapes occur at similar frequencies (Figure 8A; Appendix S1: Table S10).

Our morphological PCA and pollinator‐type analyses indicated that flower spur length strongly contributes to PC1. We reconstructed spur length across the phylogeny (Figure 7B), which revealed that species grouped within the same ASTRAL clade tend to share similar spur lengths. However, spur shape did not correspond to clade boundaries, suggesting that transitions in spur shape have occurred across different lineages of *Pinguicula* subgenus *Temnoceras*. We categorized spur shapes into three types: cylindrical, subcylindrical, and slender. Ancestral state reconstruction of spur shape was conducted using equal rates (ER), symmetric (SYM) and all rates different (ARD) Mk models, with symmetric model selected as the best fit based on AIC comparison, and the result indicated at least six independent transitions in spur shape (Figure 7D). The reconstruction suggests that the ancestral state was likely a cylindrical spur, followed by transitions to slender shapes. While cylindrical spurs are more frequent at the base of Clade 3 (highlighted in yellow), the species in this clade all possess cylindrical flower tubes and spurs—traits typically associated with short‐tongued pollinators. This clade also includes species with documented fly pollination, supporting the functional interpretation of these floral characters. Phylogenetic signal testing indicated that the symmetric (SYM) model best fits the spur shape data and that the trend from slender to subcylindrical spurs appears particularly pronounced (Figure 8B; Appendix S1: Table S10).

## DISCUSSION

### *Pinguicula* section *Temnoceras* classification

Our ASTRAL phylogenetic reconstruction, based on BUSCO gene data, included 32 species from *Pinguicula* subgenus *Temnoceras*, section *Temnoceras* sensu Fleischmann and Roccia (2018) to clarify its infrageneric classification. Based on this phylogeny, we reassigned four species to section *Temnoceras*: *P. martinezii*, *P. simulans*, *P. robertiana*, and *P. zamudioana* (Figure 2). Previously, *P. martinezii* had been placed in subgenus *Isoloba*, section *Agnata*; *P. simulans* in subgenus *Temnoceras*, section *Microphyllum*; *P. robertiana* in subgenus *Isoloba*, section *Heterophyllum*; and *P. zamudioana* in subgenus *Pinguicula*, section *Orcheosanthus* (Zamudio, 2005; Juárez‐Gutiérrez et al., 2018; Zamudio et al., 2018; Hernández‐Rendón, 2019). Our results are consistent with Fleischmann (2021), who had reassigned *P. martinezii* and *P. simulans* to section *Agnata*, and *P. robertiana* and *P. zamudioana* to section *Orcheosanthus* based on morphological traits.

We also used whole‐genome sequencing and multiple phylogenomic approaches to reconstruct the infrageneric classification of *Pinguicula* subgenus *Temnoceras* (Figure 3). According to the ASTRAL species tree, species previously assigned to Clades VII and VIII in Shimai et al. (2021) did not form monophyletic groups. Following the proposal of Fleischmann (2021), we reinterpret the “sections” defined by Shimai et al. (2021) as subsections within section Temnoceras sensu Fleischmann and Roccia (2018). In our analysis, species are grouped into (sub)section Agnata (Clades 1–3) and (sub)section Orcheosanthus (Clades 4–6), consistent with the grouping of Fleischmann (2021) (Figure 2).

Notably, the placement of *P. gypsicola* and *P. macrophylla* in our ASTRAL tree contrasts with their positions in Shimai et al. (2021), a discrepancy already noted by Fleischmann (2021). In our reconstruction, *P. gypsicola* groups with *P. emarginata* in Clade 5, placing it within (sub)section Orcheosanthus, with strong support (localPP = 1; Figure 2). In contrast, Shimai et al. (2021) placed *P. gypsicola* in Clade VIII, which Fleischmann later renamed as section *Agnata*. However, Fleischmann (2021) questioned this placement based on morphological evidence: *P. gypsicola* is morphologically more consistent with species from section *Orcheosanthus*, having “membranous, filiform summer leaves”, bilabiate flowers, funnel‐shaped tubes, and slender spurs. A similar pattern appears with *P. macrophylla*, which in our analysis falls within Clade 4 of subsection *Orcheosanthus*, though it had been assigned to Clade VIII (section *Agnata*) in Shimai et al. (2021). Interestingly, several heterophyllous *Pinguicula* species, including *P. gypsicola* (Clade 5), *P. heterophylla*, and *P. medusina* (both in Clade 6), possess linear summer leaves, despite their placement in *Orcheosanthus*, suggesting that this leaf morphology may have evolved convergently within the genus.

To investigate phylogenetic incongruence between nuclear and plastid genomes in *Pinguicula* section *Temnoceras*, we compared the BUSCO‐based ASTRAL species tree (left; Figure 3) with the plastid gene tree (right; Figure 3). While the phylogenetic backbone was largely consistent between the two, several topological conflicts were observed. Clades 1 and 3 exhibited strong congruence, whereas Clade 2 showed discordance; for example, *P. cyclosecta* and *P. kondoi* grouped with Clade 3 in the plastid tree but were placed within Clade 2 in the nuclear tree. Clades 4, 5, and 6 formed monophyletic groups in both phylogenies, however, the relationships among species within these clades varied, particularly for *P. mesophytica*, *P. parvifolia*, *P. acuminata*, and *P. crassifolia*. Compared to earlier phylogenies based on the *matK* plastid marker (Shimai et al., 2021), the plastid genome tree recovered here shows a similar overall structure. For instance, *P. miranda*, *P. conzattii*, and *P. medusina* were positioned near the root in the *matK* tree, whereas the plastome tree placed them in more derived positions, clustered with other species.

The classification proposed by Shimai et al. (2021) was based on ITS phylogenetic reconstruction, and each terminal monophyletic lineage was treated as a distinct section, resulting in the designation of Clade VII as section *Orcheosanthus* and Clade VIII as section *Agnata*. However, substantial incongruence and differences in branching order have been observed between phylogenies based on nrDNA (ITS) and cpDNA markers (*matK* and *trnL*), particularly within subgenus *Temnoceras*. In contrast, although our study also revealed some discordance between the nuclear BUSCO‐based ASTRAL species tree and the plastome tree, the overall phylogenetic backbone was consistent. Specifically, the divergence between subsections *Agnata* and *Orcheosanthus*, as well as species clustering within them, was largely congruent (Figure 3). Such incongruences are not uncommon in angiosperms and may result from processes such as incomplete lineage sorting, hybridization, or introgression (Som, 2015; Kleinkopf et al., 2019). Additionally, some species exhibited long branch lengths in the BUSCO‐based ASTRAL species tree, consistent with elevated genome‐wide substitution rates. Such heterogeneity may reflect genuine differences in substitution rates associated with genome size, repetitive content, or life history traits, although methodological factors such as hidden paralogy or errors in orthology inference could also play a role. Future work incorporating a broader sampling of *Pinguicula* species (within and outside the subgenus *Temnoceras*) and analyses such as relative rate tests would help to investigate these potential factors.

### Carnivorous plant adaptations may be tied to losses in photosynthesis‐related genes

Previous studies have reported a widespread loss of *ndh* genes from the plastid genomes of several *Pinguicula* species (Silva et al., 2018; Fu et al., 2023). However, the presence or absence of nuclear *ndh* genes in this group had not been investigated before our study. By integrating plastid and nuclear genomic data, we were able to assess *ndh* gene loss across both genomic compartments. The modified leaf morphology of carnivorous plants may reduce photosynthetic activity, potentially contributing to plastid gene loss (Wicke et al., 2014; Ross et al., 2016; Fu et al., 2023). Among carnivorous lineages with available plastome data, *Brocchinia* is the only genus reported to retain a complete plastid gene set (Fu et al., 2023). Most plastid gene losses in carnivorous plants occur within the NDH (NAD(P)H dehydrogenase‐like) complex, which functions as a plastoquinone reductase involved in ATP production, cyclic electron flow, and likely in photooxidative stress responses (Peltier et al., 2016; Yamori and Shikanai 2016; Strand et al., 2019). Despite this, the precise role of the NDH complex remains unclear, complicating efforts to explain its recurrent loss in carnivorous lineages. Loss of *ndh* genes is often an early step in plastome degradation in heterotrophic plants, many of which lack photosynthesis altogether and do not retain functional *ndh* genes (Barrett et al., 2014; Wicke et al., 2016). The succulent leaf morphology of Mexican *Pinguicula* species was described by Studnička (1991), who suggested their anatomy may resemble Kranz‐type structures typically associated with C_4_ photosynthesis. Since C_4_ carbon fixation requires more ATP than C_3_ pathways and depends heavily on the NDH complex for photosystem I‐mediated cyclic electron transport (Takabayashi et al., 2005; Ishikawa et al., 2016; Lin et al., 2017), the loss of NDH subunits in *Pinguicula* suggests that C_4_ functionality may be compromised. However, it has been proposed that carnivorous plants compensate for nutrient limitations by absorbing animal‐derived compounds, potentially reducing reliance on photosynthesis and rendering the NDH complex dispensable (Wicke et al., 2014; Fu et al., 2023). More recently, it has been shown that carnivorous plants can also assimilate carbon from their prey (Lin et al., 2025), functionally resembling heterotrophs. Given that *Pinguicula* can acquire carbon through carnivory, the degradation of photosynthesis‐related genes in both plastid and nuclear genomes may result from relaxed selective pressure to maintain their function.

### The evolution of *Pinguicula* pollination syndromes and pollinator–prey conflicts

As reproductive organs, flowers are relatively conserved structures across angiosperms, yet they exhibit remarkable diversity in traits such as architecture, color, volatiles, and pollinator rewards (Specht and Bartlett, 2009; Dellinger, 2020). Variation in floral traits among closely related species is often interpreted as an adaptation to different pollinators, whereas convergence in floral form is typically linked to parallel adaptation to the same pollinator group. Within an evolutionary framework, this reflects the idea that pollinator‐mediated selection promotes floral diversity.

Directional floral evolution driven by pollinator selection across suites of traits has been termed “pollination syndromes” (Delpino, 1874; Fenster et al., 2004; Smith et al., 2008; Dellinger, 2020). Over time, the concept of pollination syndromes has been developed and refined into a broad framework used to explain floral diversity from a functional perspective in pollination ecology (Delpino, 1874; Rosas‐Guerrero et al., 2014; Dellinger, 2020). The most recent interpretations emphasize combinations of floral traits adapted to maximize interaction with the most efficient functional pollinator group (Ashworth et al., 2015; Dellinger, 2020). Based on this framework, a comprehensive qualitative review identified 11 functional pollinator groups: bee, bird, bat, fly, wasp, moth, butterfly, long‐tongued fly, beetle, carrion fly, and non‐flying mammal (Rosas‐Guerrero et al., 2014).

*Pinguicula* exhibits a relatively conserved floral architecture but displays considerable diversity in the combination of specific floral traits across species, features that were historically used to define taxonomic groupings. Although molecular marker‐based phylogenies have refined taxonomy based on morphology, floral similarities among distantly related species suggest instances of convergence. For example, two closely related Mexican species, *P. gracilis* and *P. rotundiflora*, were initially placed in different subgenera based on floral morphology. However, recent ITS‐based phylogenetic analyses have placed both within subgenus *Temnoceras*, Clade VIII (sensu Shimai et al., 2021) and section *Agnata* (sensu Fleischmann, 2021). The observed floral divergence is thought to reflect adaptation to different local pollinators: *P. gracilis* possesses a distinctly bilabiate corolla with a large lower lip and a much smaller upper lip, while *P. rotundiflora* has a subisolobate floral form (Fleischmann, 2021).

*Pinguicula* employs a variety of pollination strategies, including myophily (fly pollination), melittophily (bee), psychophily (butterfly), and ornithophily (bird) (Lustofin et al., 2020). While clearly defined pollination syndromes are lacking in the genus, several floral traits have been consistently associated with particular pollinator groups. Psychophilous and ornithophilous species typically exhibit a combination of long nectar spurs and short, funnel‐shaped tubes, often with trichomes at the base acting as barriers to nonpollinating visitors. In contrast, myophilous and melittophilous species tend to have shorter spurs and longer, broader, usually cylindrical tubes. These flowers often bear edible trichomes near the end of the tube, just above the entrance to the spur, which functions as pollinator rewards and attract flies and bees that facilitate pollen transfer (Lustofin et al., 2023). These trichomes have been shown to vary in cell structure, secretion mode, and micromorphology, with some studies suggesting convergence among species pollinated by short‐tongued insects (Lustofin et al., 2020, 2023). Spur anatomy also displays notable diversity, and recent SEM‐based studies have uncovered multiple internal architectures potentially linked to nectar accessibility (Lustofin et al., 2020, 2023). However, edible trichomes may not be the sole or primary trait linked to fly and bee pollination. When considering the traits and pollinator data presented by Lustofin et al. (2020), as well as the topology of our ASTRAL phylogeny (Figure 2), edible trichomes may have evolved multiple times.

Studies of pollination syndromes are often restricted to narrow, qualitative trait sets rather than comprehensive quantitative analyses (Dellinger, 2020). For *Pinguicula*, relatively few floral characters have been systematically examined in relation to pollinator type (Cuevas et al., 2023; Lustofin et al., 2023). In our PCA, we expanded beyond flower tube and spur characteristics to include quantitative floral measurements, symmetry, and color. Species lacking direct pollinator records but falling into well‐defined clusters in trait space may be tentatively assigned to particular pollination systems. While speculative and requiring confirmation through field observation, this approach suggests that myophilous *Pinguicula* are morphologically distinct from ornithophilous and psychophilous species. Prior studies have shown that *Pinguicula* micromorphological floral traits align well with specific pollinator types, and our findings support this pattern.

However, it is important to note that for most species of *Pinguicula*, there is a lack of direct empirical data on pollinators, only some field observation reports of potential pollinators. For example, *P. alpina* and *P. vulgaris* were observed being visited by different species of flies (Diptera) (Molau, 1993), there was a single observation of a bumblebee queen pollinating *P. vulgaris* (Molau, 1993), and for *P. warijia*, a two‐tailed swallowtail butterfly (*Papilio multicaudata*) was observed visiting (pollinating) several flowers (Zamudio et al., 2023). Inferences about pollination syndromes are mainly based on morphological indicators. This limitation adds uncertainty to our interpretations, especially regarding ancestral state reconstructions and morphospace clustering. Recent studies in pollination ecology have highlighted that the predictive value of floral traits for pollinator identity might be weaker than previously thought, and that syndromes should be seen as hypotheses to test rather than confirmed classifications (Rosas‐Guerrero et al., 2014; Dellinger, 2020).

Psychophilous and ornithophilous *Pinguicula* species, typically bearing long spurs, funnel‐shaped tubes, and nectar trichomes within the spur, cluster together in the PCA space, as exemplified by *P. moctezumae*, *P. emarginata*, and *P. moranensis* (Figure 5; Lustofin et al., 2020). In contrast, trichomes serving as potential rewards for short‐tongued pollinators are often located at the base of the corolla tube. They are associated with fly‐ and bee‐pollinated species but are absent in butterfly‐ and bird‐pollinated taxa (Lustofin et al., 2020). Our PCA reinforces the association between phylogeny and floral morphology, suggesting that flower tube and spur structure may represent more reliable indicators of pollination syndromes in *Pinguicula* than floral color or pattern alone. Additionally, the PCA revealed that ornithophilous species share closer phylogenetic relationships with one another than with psychophilous species despite some morphological overlap. Although certain floral traits are shared by butterfly‐ and bird‐pollinated species, there remains a lack of clear morphological taxonomic criteria to consistently distinguish between these two pollination modes (Lustofin et al., 2023).

Our ancestral reconstructions of flower tube and spur traits indicate that myophilous *Pinguicula* species, such as *P. agnata*, *P. ibarrae*, *P. martinezii*, and *P. gigantea*, form a monophyletic clade along with *P. robertiana* (which has a subcylindrical flower tube). These species all share a cylindrical flower tube and cylindrical spur (Figure 7A, C), suggesting that this floral configuration is closely associated with fly pollination and may represent an adaptation driven by pollinator selection. In contrast, species such as *P. elizabethiae*, *P. moctezumae*, *P. oblongiloba*, *P. zamudioana*, and *P. macrophylla* share a combination of funnel‐shaped flower tubes and slender spurs, a trait set likely linked to butterfly pollination. However, among these, only *P. moctezumae* has been directly observed to be butterfly‐pollinated (Lustofin et al., 2020). Recently, Villegas et al. (2024) tested whether *P. moranensis* floral traits (e.g., corolla shape and spur length) are associated with female fitness under natural versus hand pollination. However, the authors only found weak support for phenotypic selection on corolla shape in naturally pollinated plants that experience pollen limitation. These findings suggest that pollen limitation could, in principle, provide conditions for pollinator‐mediated selection on floral morphology; however, additional studies are warranted for other species of *Pinguicula*.

Beyond these examples, several other clades exhibit transitions in specific floral traits. For instance, *P. heterophylla*, *P. medusina*, and *P. conzattii* possess subcylindrical spurs, even though the reconstructed ancestral state for their lineage is a slender spur. Overall, our results suggest that while certain flower characters in *Pinguicula* are significantly associated with specific pollination strategies, they do not consistently act as synapomorphies within phylogenetic clades. In addition to comparative phylogenetic analyses of morphological evolution, recent studies have explored the genetic basis of pollination syndromes, particularly genes involved in flower symmetry and spur development. For example, several investigations have implicated *STM*‐like *KNOTTED1‐LIKE* HOMEOBOX (*KNOX*) genes that are typically expressed in the shoot apical meristem in the development of floral spurs in specific plant lineages (Box et al., 2011). However, in *Aquilegia*, Yant et al. (2015) demonstrated that spur formation does not rely on extended meristematic activity or *KNOX* gene expression. Instead, early spur development was found to depend on localized cell proliferation and the expression of genes related to auxin. Although that study did not directly address the genetic architecture underlying variation in spur length, it suggests that developmental modularity may facilitate evolutionary diversification of floral structures.

Notably, the recurrence of similar floral configurations across distantly related *Pinguicula* lineages supports the idea that pollinator‐mediated selection may drive convergent evolution, a pattern that has been documented across angiosperms in quantitative syntheses of pollination syndromes and selection on floral traits, and that is often contingent on ecological context (Bartkowska and Johnson, 2012; Rosas‐Guerrero et al., 2014; Caruso et al., 2019; Sletvold, 2019). Various studies have presented evidence for the underlying genetic basis of certain morphologies that are important for pollinator attraction. For example, the *MIXTA*‐like gene *GUIDELESS* was recently found to be involved in nectar guide trichome variation among pollination systems within *Mimulus* (Chen and Yuan, 2024). Although we provide a comprehensive phylogenetic framework among *Pinguicula* subgenus *Temnoceras*, incorporating high‐quality and annotated reference genomes would further refine taxonomic resolution while also allowing for gene family studies across multiple taxa. Future research could investigate whether similar genetic modules and evolutionary transitions are responsible for pollination‐associated attributes in *Pinguicula*, potentially shedding light on the molecular mechanisms driving the evolution of pollination syndromes in this genus.

The lack of comprehensive prey and pollinator observations, as well as controlled experiments, currently limits the study of pollinator–prey conflict in carnivorous plants, including *Pinguicula*. In this study, we mainly relied on the genus‐level prey synthesis of Ellison and Gotelli (2001). A more robust analysis of prey–pollinator associations will require detailed prey records across multiple species, which could clarify whether different pollination syndromes are associated with distinct prey spectra. A recent study of *Pinguicula moranensis* found that its most frequent floral visitor belongs to the genus *Sphecodes* (Hymenoptera: Halictidae), despite the species being previously classified as psychophilous due to its bilabiate flowers, funnel‐shaped corolla tubes, and long, slender spurs (Pérez‐Alva et al., 2022). It is well‐recognized that multiple functional pollinator groups may visit plants; however, adaptations for generalist pollination are often considered costly in terms of fitness trade‐offs (Strelin et al., 2017; Dellinger, 2020). Nevertheless, evidence from *Centropogon* suggests that adaptation to multiple pollinator types can lead to the evolution of stable bimodal pollination systems (Dellinger et al., 2019). Moreover, although pollination syndromes highlight pollinators as a key source of selective pressure, other biotic interactions may also play significant roles, such as mutualistic and nonmutualistic interactions and herbivory interactions (Mithöfer, 2022). In carnivorous plants, where pollinator attraction must coexist with prey capture strategies.

When pollinators and antagonists are attracted to the same plant traits, this can create conflicting selective pressures, potentially leading to the evolution of defensive traits (Doubleday et al., 2013). In carnivorous plants, plant–insect interactions encompass not only pollination and defense but also predator–prey dynamics. Therefore, flower evolution in these plants may be shaped not only by pollinators but also by the need to manage pollinator–prey conflict. In this study, we explored this hypothesis by applying phylogenetic linear models to test for associations between traits related to carnivory and pollination, focusing on spatial strategies that may reduce overlap between prey capture and pollinator attraction. In *Pinguicula*, particularly within subgenus *Temnoceras*, most species flower during the summer, coinciding with the formation of their carnivorous rosettes. However, these species typically produce elongated scapes that elevate the flowers above the leaf rosette—a spatial separation that may function to mitigate pollinator‐prey conflict (Ellison and Gotelli, 2001).

Our ancestral state reconstruction indicates a trend toward longer scapes, while phylogenetic linear models reveal a marginally significant association between scape length and carnivorous leaf area. Longer scapes, or increased inflorescence height, can provide reproductive advantages such as enhanced pollinator visitation and broader seed dispersal (Ågren et al., 2006; Chen and Pannell, 2022). However, the marginal correlation we observed (*P* = 0.05722; Appendix S1: Table S8) suggests that in *Pinguicula*, scape elongation may be more strongly influenced by allometric scaling than by selection to reduce pollinator–prey conflict. A similar pattern has been noted in the carnivorous genus *Drosera*, where longer scapes may increase pollinator attraction, though not necessarily as a mechanism to reduce pollinator–prey conflict (Anderson, 2010). Experimental work in *Drosera* further indicates that spatial separation and multimodal divergence between flowers and traps can reduce unintended pollinator capture, supporting the adaptive value of spatial segregation in mitigating pollinator–prey conflict (Jürgens et al., 2015; El‐Sayed et al., 2016). In *Dionaea muscipula*, field evidence similarly shows near‐zero overlap between flower visitors and trapped prey, consistent with a role for vertical separation between flowers and traps (Youngsteadt et al., 2018). Beyond plant morphology, pollinator behavior itself may play a crucial role in modulating the intensity of this conflict. Gavini and Quintero (2024) proposed a risk–reward foraging framework in which pollinator foraging can decline sharply with increasing predation risk when floral rewards increase. Furthermore, pollinators may use visual or olfactory cues to determine the risk of predation. These characteristics of plant, pollinator, and predator interactions underscore the importance of integrating both ecological context and pollinator decision‐making into interpretations of floral trait evolution in carnivorous lineages.

In *Pinguicula*, particularly within subsection *Orcheosanthus*, several species exhibit floral traits consistent with specialization for butterfly and hummingbird pollination, and field observations support these associations (Pérez‐Alva et al., 2022). The evolutionary transition from short‐tongued to long‐tongued pollinators is expected based on spur morphology, though further observational data are needed to confirm this trend.

In *Silene*, hummingbirds have been shown to prefer higher inflorescences (Fenster et al., 2015), a pattern that mirrors the trend observed in *Pinguicula* scape elongation. Additionally, numerous plant lineages exhibit evolutionary transitions from insect to bird pollination (Navarro‐Pérez et al., 2013), suggesting that pollinator shifts, rather than pollinator–prey conflict alone, may be a primary driver behind increased floral height.

Our results reveal a significant coordinated evolutionary pattern between scape length and carnivorous leaf area, suggesting that plant carnivory may have influenced the spatial separation of these structures, likely as part of strategies to reduce pollinator–prey conflict. However, our analysis focused on a single pollinator–prey conflict scenario (scape length vs. trap area), and other traits contributing to spatial, temporal or functional separation may also be important. In our data set, several *Pinguicula* species (e.g., *P. kondoi*, *P. laueana*, *P. rotundiflora*) flower during the winter rosette stage, which is commonly regarded as noncarnivorous. This flowering habit may represent a temporal strategy to reduce pollinator–prey conflict. For these species, we used winter leaf measurements in the analyses to maintain statistical power and because the potential carnivorous function of winter rosettes has not been empirically excluded. Nonetheless, the role of temporal strategies in avoiding pollinator–prey conflict remains to be tested explicitly, which will require more comprehensive pollinator observations and a broader phylogenetic framework.

As for the functional separation, one such possibility involves the emission of distinct volatile organic compound (VOC) profiles by flowers and traps (Tagawa, 2020). Several carnivorous species in the Caryophyllales and Ericales are known to produce different VOCs to attract either pollinators or prey (El‐Sayed et al., 2016; Ho et al., 2017; Tagawa, 2020; Ojeda et al., 2021; Tagawa et al., 2024). In *Pinguicula*, relatively few studies have addressed whether floral and trap volatiles are differentiated in this way. In *P. moranensis*, for example, skipper butterflies (used as a pollinator model) preferred floral scents over clean air, while *Drosophila melanogaster* (used as a prey model) was more strongly attracted to leaf‐trap scents (Cuevas et al., 2023). However, the dipteran model did not show a statistically significant difference in preference between floral and trap volatiles, suggesting that, at least in *P. moranensis*, floral VOCs may not be under strong selective pressure to exclude nonpollinators and instead may act as general attractants for both pollinators and prey.

Future studies should integrate multiple traits, such as scape length, trap size, and VOC composition, across carnivorous species and their noncarnivorous relatives, using multivariate frameworks that account for allometric scaling. This approach could help disentangle the evolutionary trajectories of traits shaped by pollinator–prey conflict from those driven solely by pollinator‐mediated selection.

## CONCLUSIONS

Our study presents a robust and well‐resolved genome‐wide phylogeny for *Pinguicula*, refining the infrageneric classification of section *Temnoceras* and providing key updates to species placements, such as the reassignment of *P. gypsicola* and *P. macrophylla*. Our integrative phylogenetic analyses yielded multiple insights into floral morphological evolution, including strong clustering of traits by pollination strategy and evidence of repeated transitions in spur and tube morphology, as revealed through ancestral state reconstruction.

We also documented widespread loss or pseudogenization of *ndh* genes in both nuclear and plastid genomes, potentially affecting photosynthetic function. Additionally, we observed a correlation between scape length and carnivorous leaf area, suggesting a functional link between spatial floral positioning and the ecological trade‐off of attracting pollinators while capturing insect prey.

Together, these findings underscore the dual roles of pollinator specialization and carnivory‐related traits in shaping floral evolution. However, recent phylogenomic studies in *Mimulus* challenge the assumption that trait similarity always results from convergence. Earlier AFLP‐based studies supported the repeated independent origins of hummingbird‐pollinated traits in *Erythranthe*, which is viewed as a model for rapid speciation through pollination shifts (Beardsley et al., 2003). In contrast, genome‐wide analyses have revealed that these traits likely originated from a shared ancestor and were shaped by both ancient and recent introgression (Nelson et al., 2021). These findings underscore the significance of genome‐scale data in understanding trait evolution, even in well‐established model systems.

In this study, we employed genome‐wide BUSCO gene sampling to reconstruct the phylogeny of the *Pinguicula* subgenus *Temnoceras*, offering higher resolution than previous marker‐limited approaches. Beyond the current analyses, the genome sequencing data, nuclear genome assemblies, and plastome assemblies generated here represent a valuable resource for broader studies in population and evolutionary genetics. These resources could be used in SNP‐based analyses, for *D*‐statistics, or phylogenetic network approaches to investigate processes such as incomplete lineage sorting, hybridization, and introgression. However, unlike the *Mimulus* example, we did not investigate regional introgression or gene tree discordance. As high‐quality reference genomes for *Pinguicula* become available, future research could utilize reference‐guided genome scans to determine whether similarities in floral traits reflect true convergence or, instead, result from shared ancestry and gene flow. Such studies will be critical for fully resolving the evolutionary history of pollination syndromes in *Pinguicula*. Future work should focus on direct observational and experimental studies of pollinator identity and behavior to validate the trait‐based hypotheses proposed here.

## AUTHOR CONTRIBUTIONS

Y.L. and T.R. conceived of the project. M.M.‐R. and E.I.‐L. provided plant tissue. Y.L. performed DNA extractions, collected morphological data, assembled and analyzed DNAseq data, investigated nuclear *ndh* gene loss, and conducted phylogenetic analyses. Q.L. conducted analyses of chloroplast *ndh* gene loss. S.J.F. provided guidance on genome assembly and reconstruction of the ASTRAL consensus tree. Y.L., Q.L., and T.R. interpreted the results. Y.L. prepared the figures. Y.L., Q.L., and T.R. wrote the initial draft of the manuscript. Y.L, Q.L., S.J.F., M.M.‐R., E.I.‐L., and T.R. provided comments on the draft and made final edits to the manuscript. T.R. contributed funding for the project. We thank the Associate Editor and reviewers for their constructive comments and suggestions that improved this manuscript.

## Supporting information


**Appendix S1.** Tables S1–S10.
**Table S1.** *Pinguicula* specimen information.
**Table S2.** *Pinguicula* plastid genes and nuclear *ndh* gene query.
**Table S3.** Morphological data collection for *Pinguicula* species.
**Table S4.** Quality summary of Illumina sequencing reads.
**Table S5.** Quality summary of genome assemblies.
**Table S6.** Summary of estimated *Pinguicula* ploidy level.
**Table S7.** Missing BUSCOs.
**Table S8.** Phylogenetic linear model analyses.
**Table S9.** Phylogenetic ancestral state reconstruction analyses.
**Table S10.** *Pinguicula* flower character transition model fitting test.

**Appendix S2.** Smudgeplots for 32 *Pinguicula* species, each showing the proposed ploidy level and the *k*‐mer pair coverage used to estimate genome characteristics.

**Appendix S3.** Shell scripts containing the full code of extracting BUSCO genes from genome assemblies, aligning, trimming, building gene phylogenetic reconstruction and final species phylogenetic reconstruction, adding branch lengths based on substitution rates. BUSCO alignment input for IQ TREE gene trees and ASTRAL consensus species tree, along with the resulting tree files, are included.

**Appendix S4.** Alignment of genes for plastid gene tree and resulting tree file.

**Appendix S5.** R Markdown file containing the full code and output for principal component analysis (PCA), phylogenetic linear models, and ancestral state reconstruction tests, along with the corresponding figures and statistical results.

## Data Availability

Illumina reads to be released on the NCBI Sequence Read Archive (SRA) upon acceptance under BioProject PRJNA1305498 (http://www.ncbi.nlm.nih.gov/sra). Nuclear and plastid genome assemblies are available on The Pennsylvania State University's ScholarSphere (https://doi.org/10.26207/e27k-mv04).
