# Supplementary figures and images for: Phylogenomics reveals the evolution of floral traits associated with pollinators and pollinator–prey conflict within the carnivorous Pinguicula subgenus Temnoceras
Source: Am J Bot. 2026 Jan 31;113(2):e70156. doi: 10.1002/ajb2.70156 (PMC12918848; doi:10.1002/ajb2.70156)

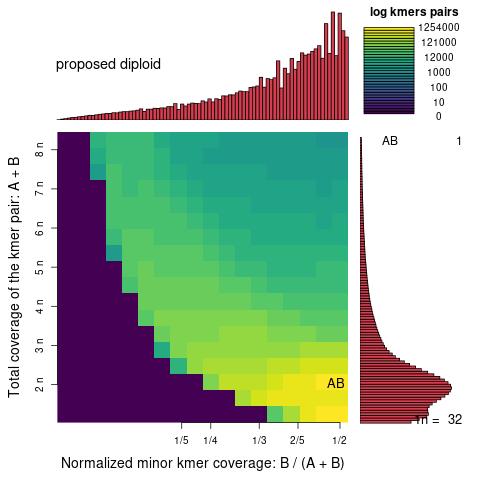

Supplement: Supplementary file 2 — Appendix S2. Smudgeplots for 32 Pinguicula species, each showing the proposed ploidy level and the k‐mer pair coverage used to estimate genome characteristics. [file AJB2-113-e70156-s005.zip › AppendixS2/P27_1.Pinguicula_moranensis.smudgeplot_smudgeplot_log10.png]

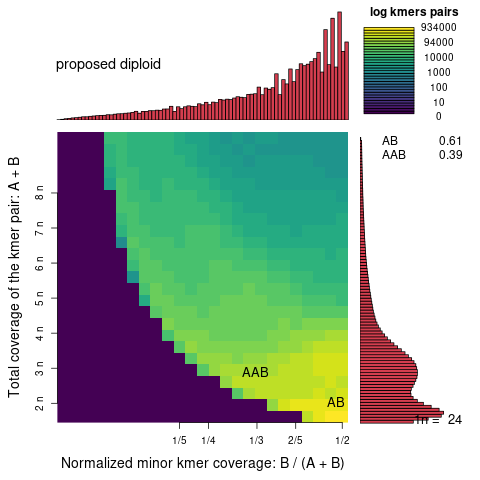

Supplement: Supplementary file 2 — Appendix S2. Smudgeplots for 32 Pinguicula species, each showing the proposed ploidy level and the k‐mer pair coverage used to estimate genome characteristics. [file AJB2-113-e70156-s005.zip › AppendixS2/P31_1.Pinguicula_parvifolia.smudgeplot_smudgeplot_log10.png]

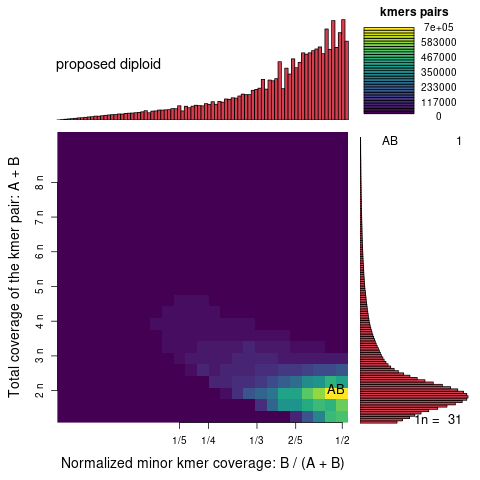

Supplement: Supplementary file 2 — Appendix S2. Smudgeplots for 32 Pinguicula species, each showing the proposed ploidy level and the k‐mer pair coverage used to estimate genome characteristics. [file AJB2-113-e70156-s005.zip › AppendixS2/P03_1.Pinguicula_caerulea.smudgeplot_smudgeplot.png]

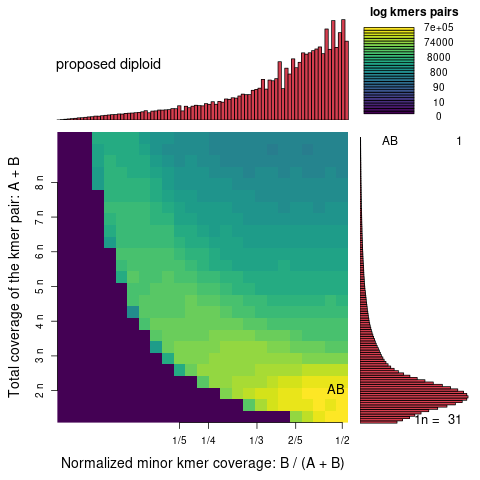

Supplement: Supplementary file 2 — Appendix S2. Smudgeplots for 32 Pinguicula species, each showing the proposed ploidy level and the k‐mer pair coverage used to estimate genome characteristics. [file AJB2-113-e70156-s005.zip › AppendixS2/P03_1.Pinguicula_caerulea.smudgeplot_smudgeplot_log10.png]

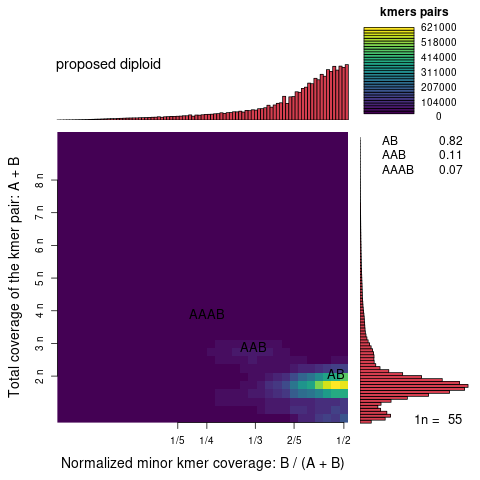

Supplement: Supplementary file 2 — Appendix S2. Smudgeplots for 32 Pinguicula species, each showing the proposed ploidy level and the k‐mer pair coverage used to estimate genome characteristics. [file AJB2-113-e70156-s005.zip › AppendixS2/P16.Pinguicula_heterophylla.smudgeplot_smudgeplot.png]

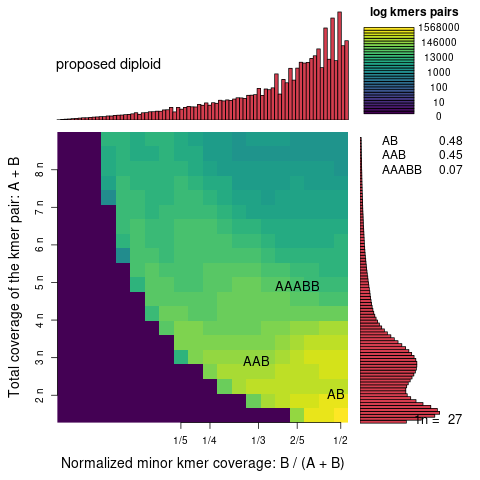

Supplement: Supplementary file 2 — Appendix S2. Smudgeplots for 32 Pinguicula species, each showing the proposed ploidy level and the k‐mer pair coverage used to estimate genome characteristics. [file AJB2-113-e70156-s005.zip › AppendixS2/P32_1.Pinguicula_rectifolia.smudgeplot_smudgeplot_log10.png]

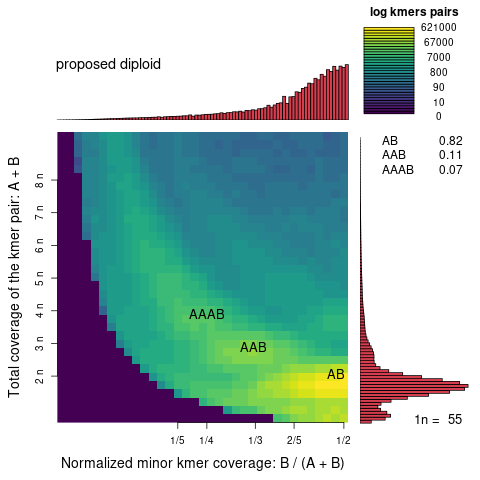

Supplement: Supplementary file 2 — Appendix S2. Smudgeplots for 32 Pinguicula species, each showing the proposed ploidy level and the k‐mer pair coverage used to estimate genome characteristics. [file AJB2-113-e70156-s005.zip › AppendixS2/P16.Pinguicula_heterophylla.smudgeplot_smudgeplot_log10.png]

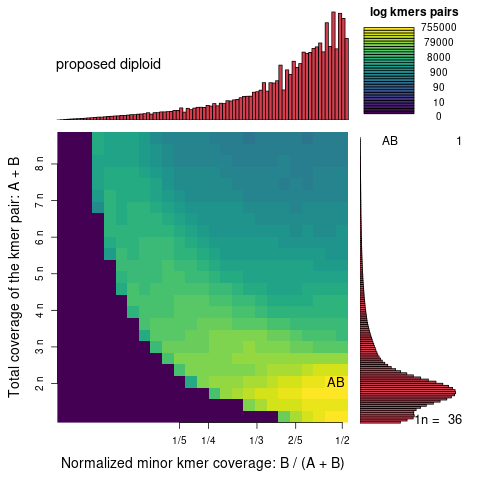

Supplement: Supplementary file 2 — Appendix S2. Smudgeplots for 32 Pinguicula species, each showing the proposed ploidy level and the k‐mer pair coverage used to estimate genome characteristics. [file AJB2-113-e70156-s005.zip › AppendixS2/P11_1.Pinguicula_elizabethiae.smudgeplot_smudgeplot_log10.png]

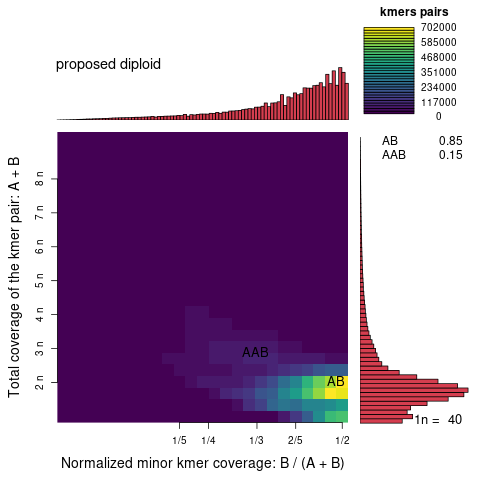

Supplement: Supplementary file 2 — Appendix S2. Smudgeplots for 32 Pinguicula species, each showing the proposed ploidy level and the k‐mer pair coverage used to estimate genome characteristics. [file AJB2-113-e70156-s005.zip › AppendixS2/P37.Pinguicula_mesophytica.smudgeplot_smudgeplot.png]

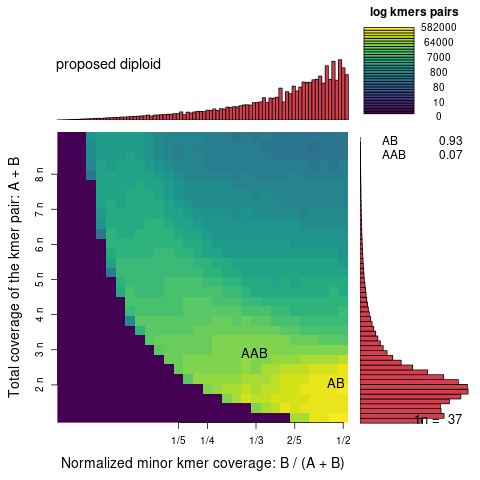

Supplement: Supplementary file 2 — Appendix S2. Smudgeplots for 32 Pinguicula species, each showing the proposed ploidy level and the k‐mer pair coverage used to estimate genome characteristics. [file AJB2-113-e70156-s005.zip › AppendixS2/P35.Pinguicula_simulans.smudgeplot_smudgeplot_log10.png]

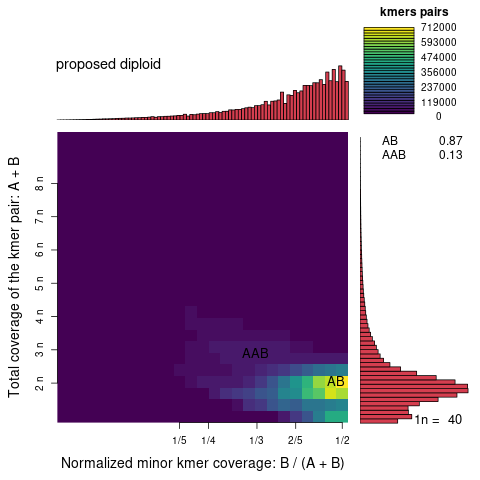

Supplement: Supplementary file 2 — Appendix S2. Smudgeplots for 32 Pinguicula species, each showing the proposed ploidy level and the k‐mer pair coverage used to estimate genome characteristics. [file AJB2-113-e70156-s005.zip › AppendixS2/P18.Pinguicula_immaculata.smudgeplot_smudgeplot.png]

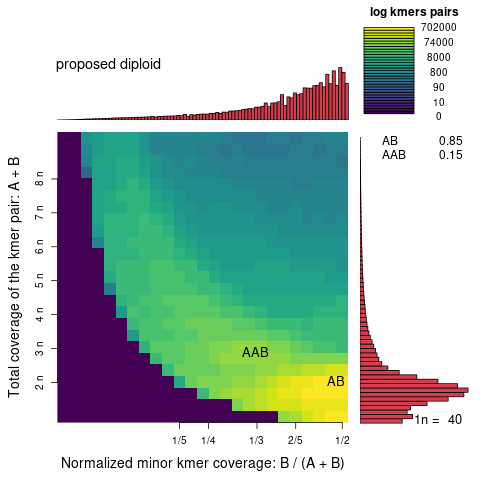

Supplement: Supplementary file 2 — Appendix S2. Smudgeplots for 32 Pinguicula species, each showing the proposed ploidy level and the k‐mer pair coverage used to estimate genome characteristics. [file AJB2-113-e70156-s005.zip › AppendixS2/P37.Pinguicula_mesophytica.smudgeplot_smudgeplot_log10.png]

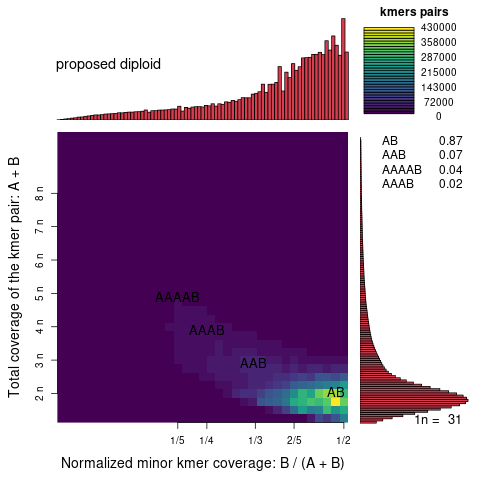

Supplement: Supplementary file 2 — Appendix S2. Smudgeplots for 32 Pinguicula species, each showing the proposed ploidy level and the k‐mer pair coverage used to estimate genome characteristics. [file AJB2-113-e70156-s005.zip › AppendixS2/P30_1.Pinguicula_orchidioides.smudgeplot_smudgeplot.png]

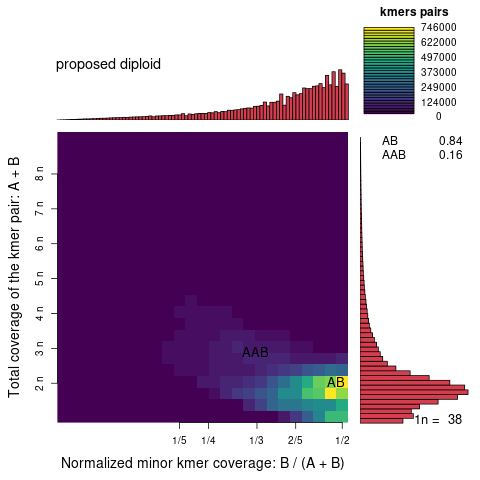

Supplement: Supplementary file 2 — Appendix S2. Smudgeplots for 32 Pinguicula species, each showing the proposed ploidy level and the k‐mer pair coverage used to estimate genome characteristics. [file AJB2-113-e70156-s005.zip › AppendixS2/P01_1.Pinguicula_acuminata.smudgeplot_smudgeplot.png]

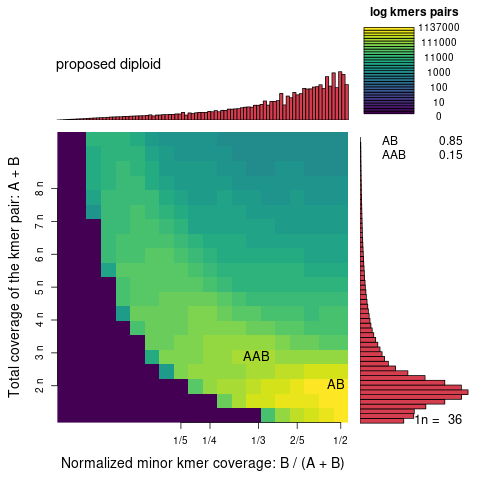

Supplement: Supplementary file 2 — Appendix S2. Smudgeplots for 32 Pinguicula species, each showing the proposed ploidy level and the k‐mer pair coverage used to estimate genome characteristics. [file AJB2-113-e70156-s005.zip › AppendixS2/P15_1.Pinguicula_hemiepiphytica.smudgeplot_smudgeplot_log10.png]

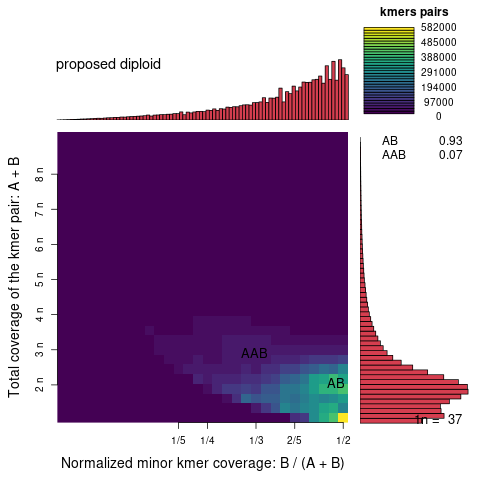

Supplement: Supplementary file 2 — Appendix S2. Smudgeplots for 32 Pinguicula species, each showing the proposed ploidy level and the k‐mer pair coverage used to estimate genome characteristics. [file AJB2-113-e70156-s005.zip › AppendixS2/P35.Pinguicula_simulans.smudgeplot_smudgeplot.png]

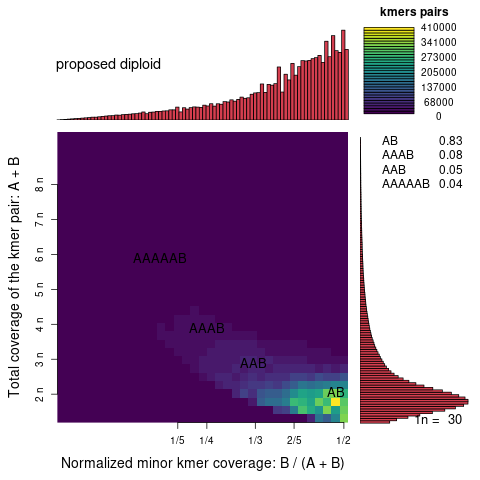

Supplement: Supplementary file 2 — Appendix S2. Smudgeplots for 32 Pinguicula species, each showing the proposed ploidy level and the k‐mer pair coverage used to estimate genome characteristics. [file AJB2-113-e70156-s005.zip › AppendixS2/P21_1.Pinguicula_laxifolia.smudgeplot_smudgeplot.png]

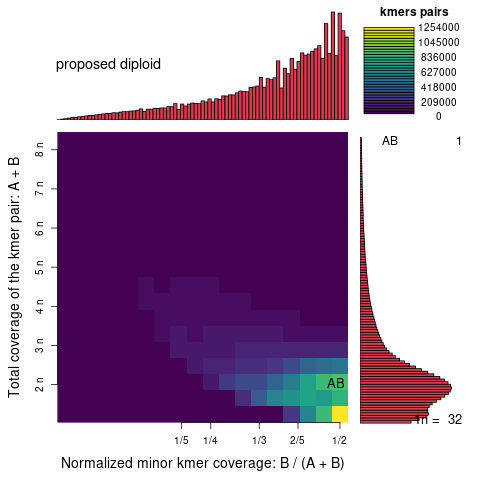

Supplement: Supplementary file 2 — Appendix S2. Smudgeplots for 32 Pinguicula species, each showing the proposed ploidy level and the k‐mer pair coverage used to estimate genome characteristics. [file AJB2-113-e70156-s005.zip › AppendixS2/P27_1.Pinguicula_moranensis.smudgeplot_smudgeplot.png]

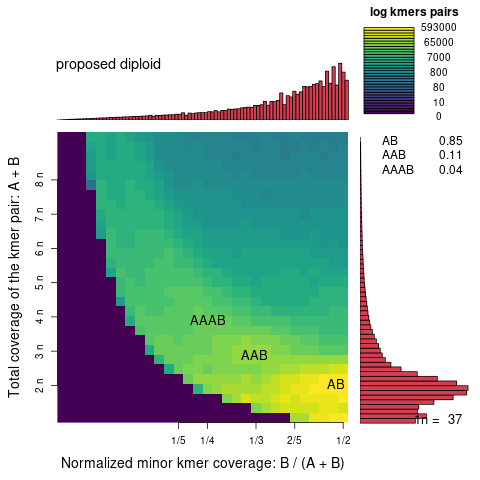

Supplement: Supplementary file 2 — Appendix S2. Smudgeplots for 32 Pinguicula species, each showing the proposed ploidy level and the k‐mer pair coverage used to estimate genome characteristics. [file AJB2-113-e70156-s005.zip › AppendixS2/P22.Pinguicula_macrophylla.smudgeplot_smudgeplot_log10.png]

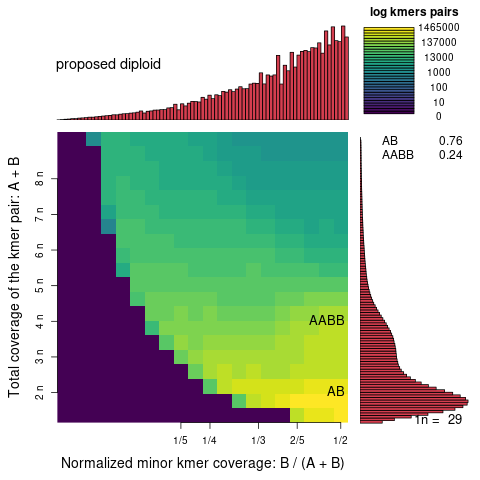

Supplement: Supplementary file 2 — Appendix S2. Smudgeplots for 32 Pinguicula species, each showing the proposed ploidy level and the k‐mer pair coverage used to estimate genome characteristics. [file AJB2-113-e70156-s005.zip › AppendixS2/P10.Pinguicula_ehlersiae.smudgeplot_smudgeplot_log10.png]

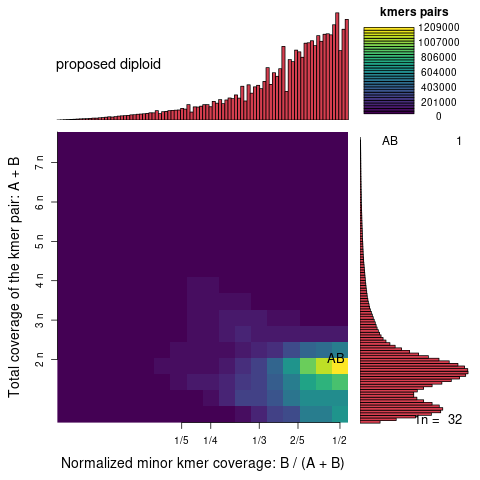

Supplement: Supplementary file 2 — Appendix S2. Smudgeplots for 32 Pinguicula species, each showing the proposed ploidy level and the k‐mer pair coverage used to estimate genome characteristics. [file AJB2-113-e70156-s005.zip › AppendixS2/P06_1.Pinguicula_conzattii.smudgeplot_smudgeplot.png]

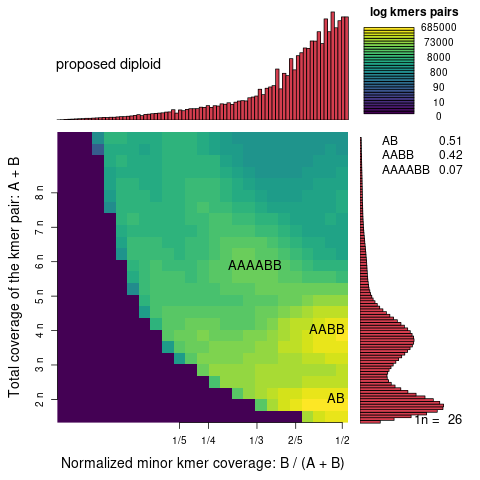

Supplement: Supplementary file 2 — Appendix S2. Smudgeplots for 32 Pinguicula species, each showing the proposed ploidy level and the k‐mer pair coverage used to estimate genome characteristics. [file AJB2-113-e70156-s005.zip › AppendixS2/P24.Pinguicula_medusina.smudgeplot_smudgeplot_log10.png]

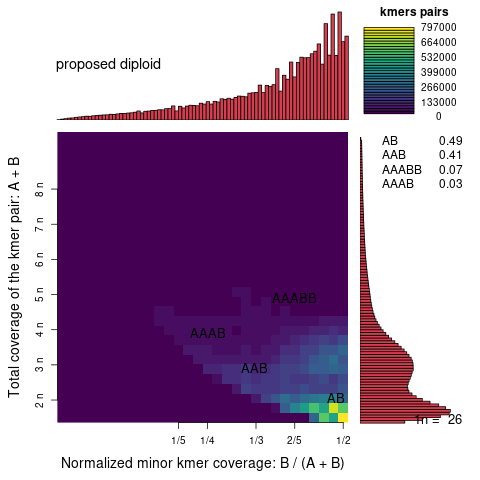

Supplement: Supplementary file 2 — Appendix S2. Smudgeplots for 32 Pinguicula species, each showing the proposed ploidy level and the k‐mer pair coverage used to estimate genome characteristics. [file AJB2-113-e70156-s005.zip › AppendixS2/PEM.Pinguicula_emarginata.smudgeplot_smudgeplot.png]

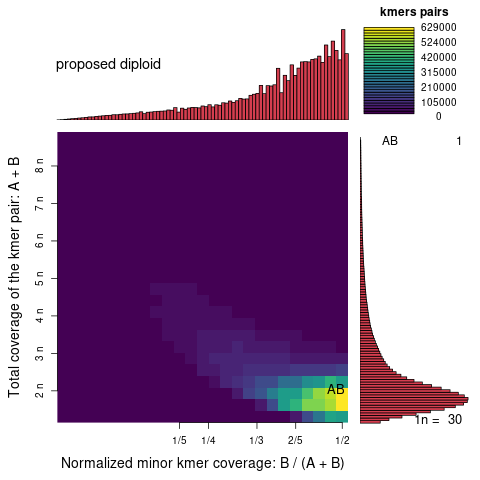

Supplement: Supplementary file 2 — Appendix S2. Smudgeplots for 32 Pinguicula species, each showing the proposed ploidy level and the k‐mer pair coverage used to estimate genome characteristics. [file AJB2-113-e70156-s005.zip › AppendixS2/P07_1.Pinguicula_crassifolia.smudgeplot_smudgeplot.png]

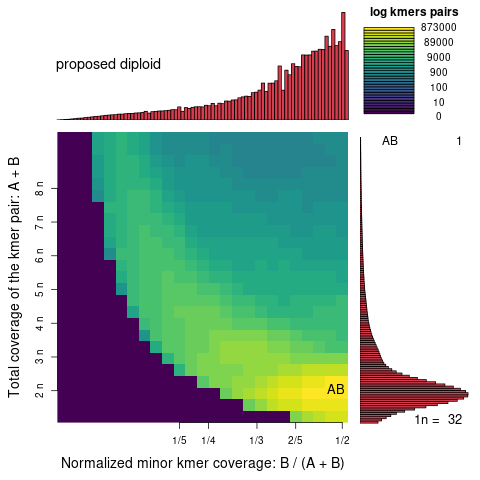

Supplement: Supplementary file 2 — Appendix S2. Smudgeplots for 32 Pinguicula species, each showing the proposed ploidy level and the k‐mer pair coverage used to estimate genome characteristics. [file AJB2-113-e70156-s005.zip › AppendixS2/P17.Pinguicula_ibarrae.smudgeplot_smudgeplot_log10.png]

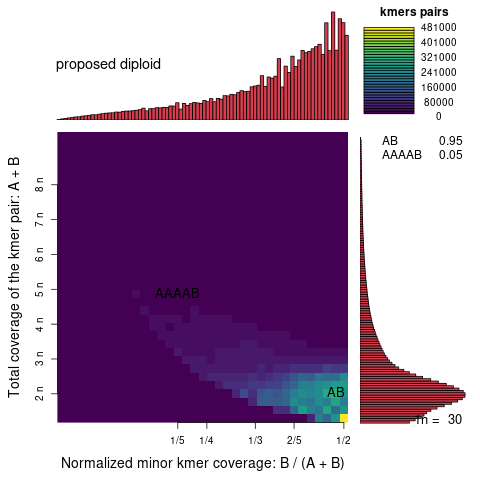

Supplement: Supplementary file 2 — Appendix S2. Smudgeplots for 32 Pinguicula species, each showing the proposed ploidy level and the k‐mer pair coverage used to estimate genome characteristics. [file AJB2-113-e70156-s005.zip › AppendixS2/P20_1.Pinguicula_laueana.smudgeplot_smudgeplot.png]

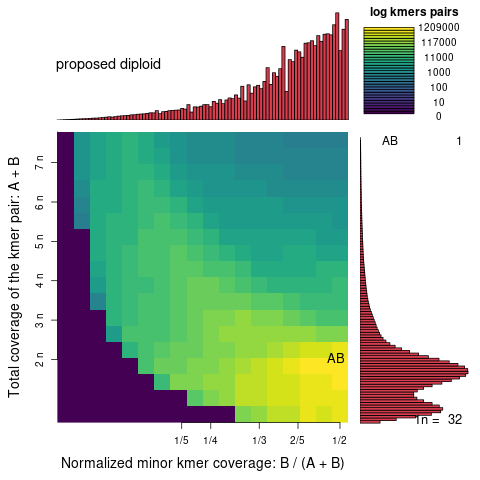

Supplement: Supplementary file 2 — Appendix S2. Smudgeplots for 32 Pinguicula species, each showing the proposed ploidy level and the k‐mer pair coverage used to estimate genome characteristics. [file AJB2-113-e70156-s005.zip › AppendixS2/P06_1.Pinguicula_conzattii.smudgeplot_smudgeplot_log10.png]

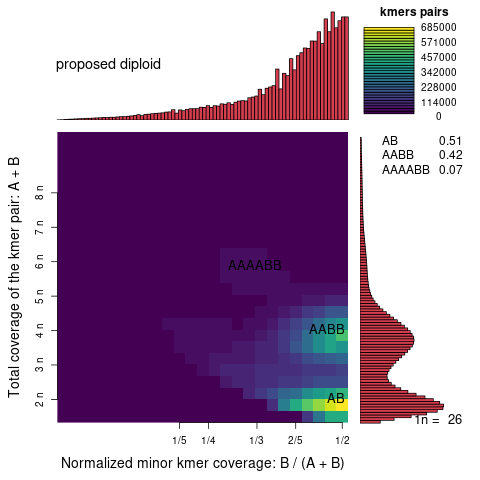

Supplement: Supplementary file 2 — Appendix S2. Smudgeplots for 32 Pinguicula species, each showing the proposed ploidy level and the k‐mer pair coverage used to estimate genome characteristics. [file AJB2-113-e70156-s005.zip › AppendixS2/P24.Pinguicula_medusina.smudgeplot_smudgeplot.png]

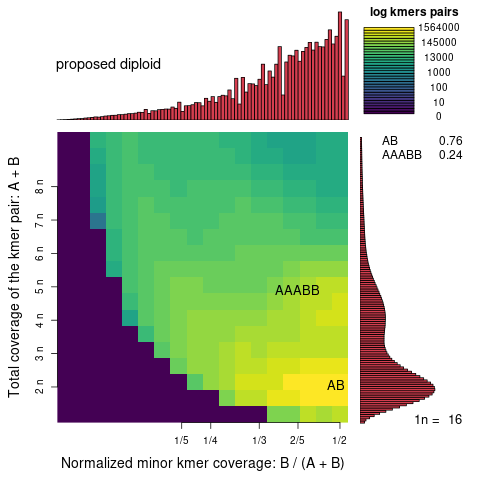

Supplement: Supplementary file 2 — Appendix S2. Smudgeplots for 32 Pinguicula species, each showing the proposed ploidy level and the k‐mer pair coverage used to estimate genome characteristics. [file AJB2-113-e70156-s005.zip › AppendixS2/P23.Pinguicula_martinezii.smudgeplot_smudgeplot_log10.png]

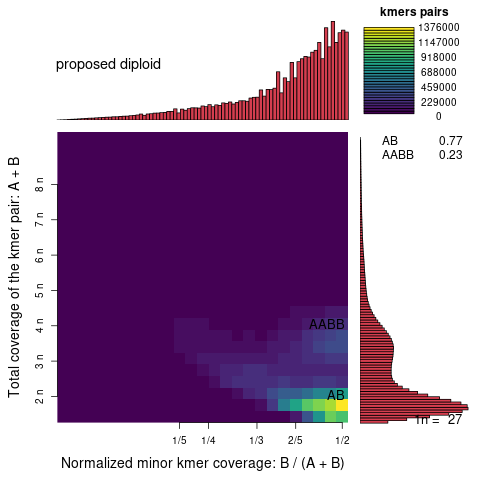

Supplement: Supplementary file 2 — Appendix S2. Smudgeplots for 32 Pinguicula species, each showing the proposed ploidy level and the k‐mer pair coverage used to estimate genome characteristics. [file AJB2-113-e70156-s005.zip › AppendixS2/P09.Pinguicula_debbertiana.smudgeplot_smudgeplot.png]

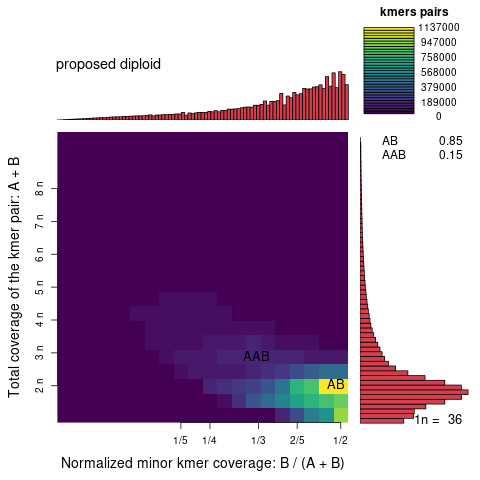

Supplement: Supplementary file 2 — Appendix S2. Smudgeplots for 32 Pinguicula species, each showing the proposed ploidy level and the k‐mer pair coverage used to estimate genome characteristics. [file AJB2-113-e70156-s005.zip › AppendixS2/P15_1.Pinguicula_hemiepiphytica.smudgeplot_smudgeplot.png]

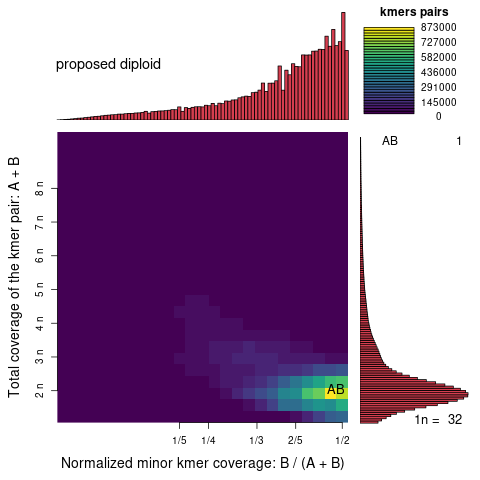

Supplement: Supplementary file 2 — Appendix S2. Smudgeplots for 32 Pinguicula species, each showing the proposed ploidy level and the k‐mer pair coverage used to estimate genome characteristics. [file AJB2-113-e70156-s005.zip › AppendixS2/P17.Pinguicula_ibarrae.smudgeplot_smudgeplot.png]

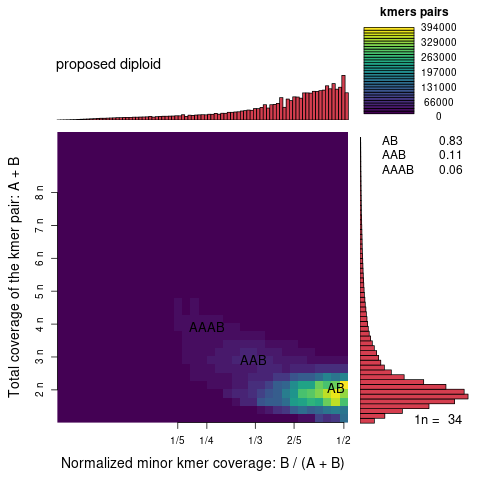

Supplement: Supplementary file 2 — Appendix S2. Smudgeplots for 32 Pinguicula species, each showing the proposed ploidy level and the k‐mer pair coverage used to estimate genome characteristics. [file AJB2-113-e70156-s005.zip › AppendixS2/P36_1.Pinguicula_zamudioana.smudgeplot_smudgeplot.png]

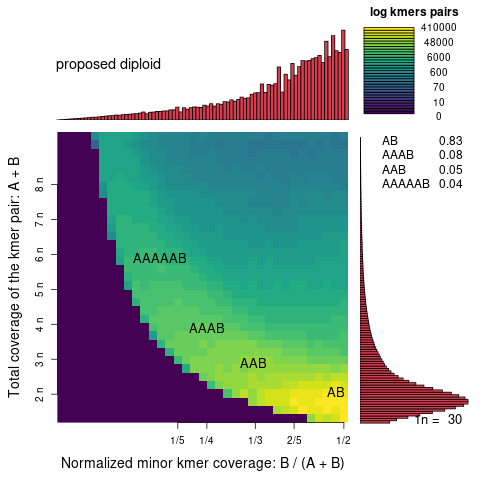

Supplement: Supplementary file 2 — Appendix S2. Smudgeplots for 32 Pinguicula species, each showing the proposed ploidy level and the k‐mer pair coverage used to estimate genome characteristics. [file AJB2-113-e70156-s005.zip › AppendixS2/P21_1.Pinguicula_laxifolia.smudgeplot_smudgeplot_log10.png]

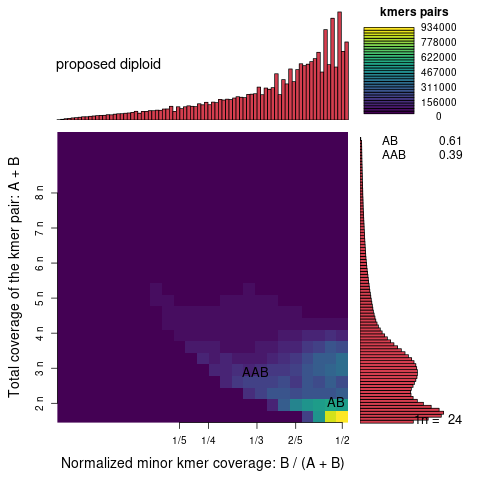

Supplement: Supplementary file 2 — Appendix S2. Smudgeplots for 32 Pinguicula species, each showing the proposed ploidy level and the k‐mer pair coverage used to estimate genome characteristics. [file AJB2-113-e70156-s005.zip › AppendixS2/P31_1.Pinguicula_parvifolia.smudgeplot_smudgeplot.png]

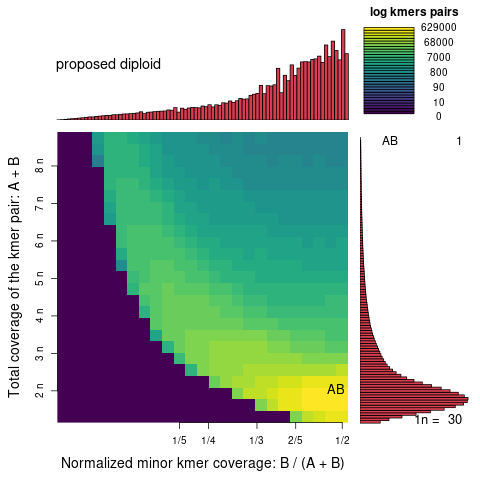

Supplement: Supplementary file 2 — Appendix S2. Smudgeplots for 32 Pinguicula species, each showing the proposed ploidy level and the k‐mer pair coverage used to estimate genome characteristics. [file AJB2-113-e70156-s005.zip › AppendixS2/P07_1.Pinguicula_crassifolia.smudgeplot_smudgeplot_log10.png]

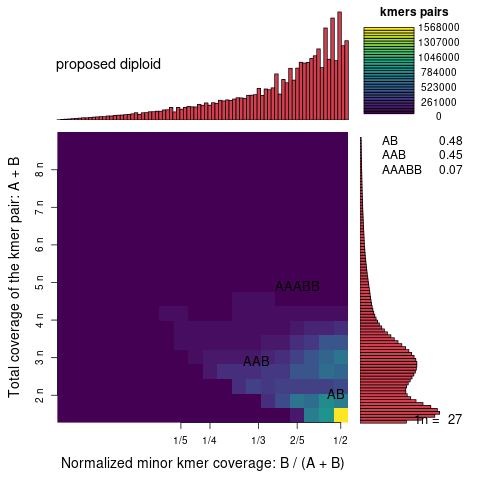

Supplement: Supplementary file 2 — Appendix S2. Smudgeplots for 32 Pinguicula species, each showing the proposed ploidy level and the k‐mer pair coverage used to estimate genome characteristics. [file AJB2-113-e70156-s005.zip › AppendixS2/P32_1.Pinguicula_rectifolia.smudgeplot_smudgeplot.png]

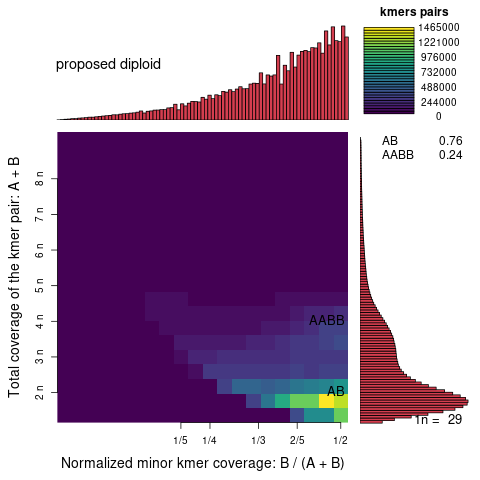

Supplement: Supplementary file 2 — Appendix S2. Smudgeplots for 32 Pinguicula species, each showing the proposed ploidy level and the k‐mer pair coverage used to estimate genome characteristics. [file AJB2-113-e70156-s005.zip › AppendixS2/P10.Pinguicula_ehlersiae.smudgeplot_smudgeplot.png]

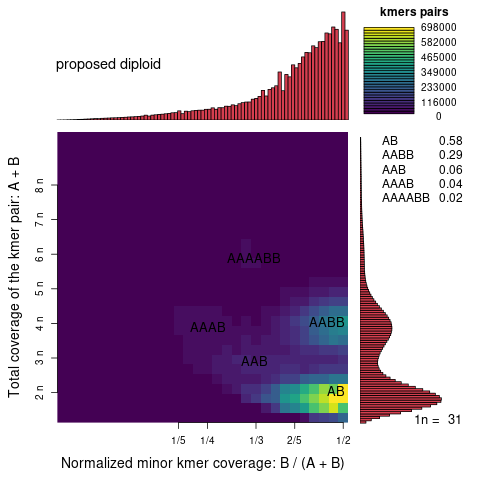

Supplement: Supplementary file 2 — Appendix S2. Smudgeplots for 32 Pinguicula species, each showing the proposed ploidy level and the k‐mer pair coverage used to estimate genome characteristics. [file AJB2-113-e70156-s005.zip › AppendixS2/P08.Pinguicula_cyclosecta.smudgeplot_smudgeplot.png]

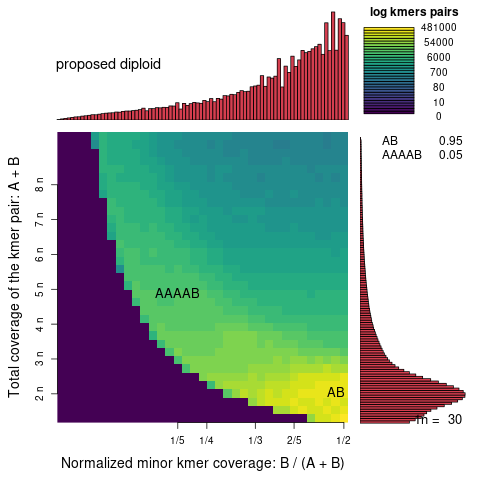

Supplement: Supplementary file 2 — Appendix S2. Smudgeplots for 32 Pinguicula species, each showing the proposed ploidy level and the k‐mer pair coverage used to estimate genome characteristics. [file AJB2-113-e70156-s005.zip › AppendixS2/P20_1.Pinguicula_laueana.smudgeplot_smudgeplot_log10.png]

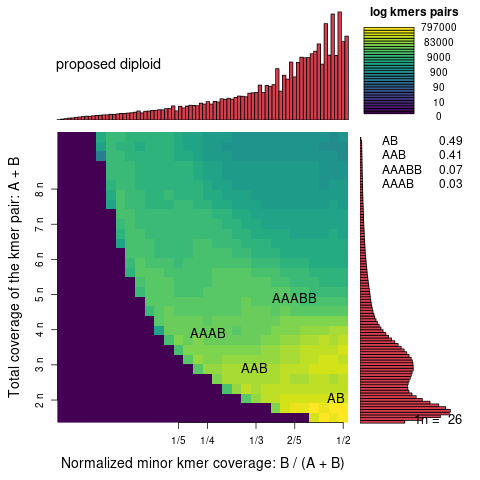

Supplement: Supplementary file 2 — Appendix S2. Smudgeplots for 32 Pinguicula species, each showing the proposed ploidy level and the k‐mer pair coverage used to estimate genome characteristics. [file AJB2-113-e70156-s005.zip › AppendixS2/PEM.Pinguicula_emarginata.smudgeplot_smudgeplot_log10.png]

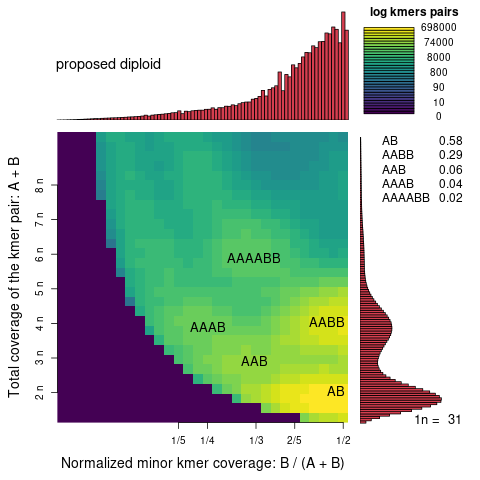

Supplement: Supplementary file 2 — Appendix S2. Smudgeplots for 32 Pinguicula species, each showing the proposed ploidy level and the k‐mer pair coverage used to estimate genome characteristics. [file AJB2-113-e70156-s005.zip › AppendixS2/P08.Pinguicula_cyclosecta.smudgeplot_smudgeplot_log10.png]

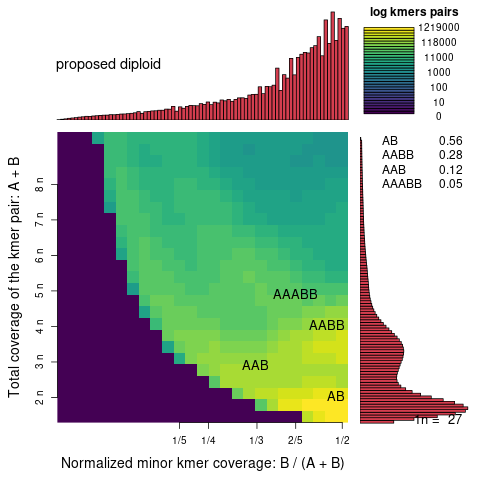

Supplement: Supplementary file 2 — Appendix S2. Smudgeplots for 32 Pinguicula species, each showing the proposed ploidy level and the k‐mer pair coverage used to estimate genome characteristics. [file AJB2-113-e70156-s005.zip › AppendixS2/P14.Pinguicula_gypsicola.smudgeplot_smudgeplot_log10.png]

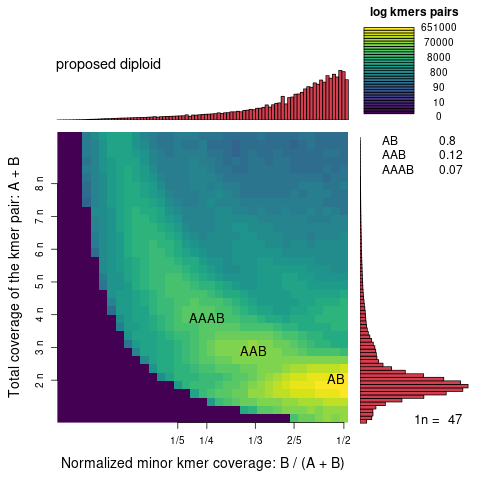

Supplement: Supplementary file 2 — Appendix S2. Smudgeplots for 32 Pinguicula species, each showing the proposed ploidy level and the k‐mer pair coverage used to estimate genome characteristics. [file AJB2-113-e70156-s005.zip › AppendixS2/P26_1.Pinguicula_moctezumae.smudgeplot_smudgeplot_log10.png]

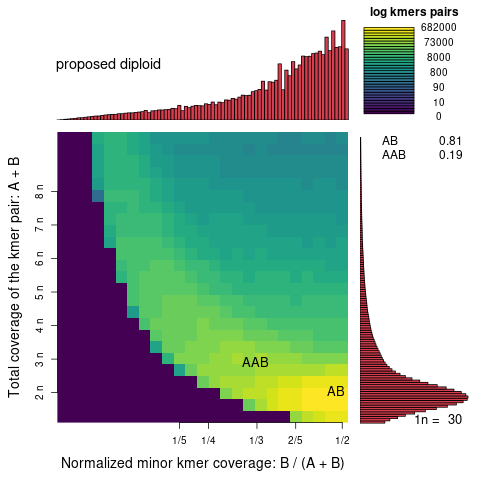

Supplement: Supplementary file 2 — Appendix S2. Smudgeplots for 32 Pinguicula species, each showing the proposed ploidy level and the k‐mer pair coverage used to estimate genome characteristics. [file AJB2-113-e70156-s005.zip › AppendixS2/P33_1.Pinguicula_robertiana.smudgeplot_smudgeplot_log10.png]

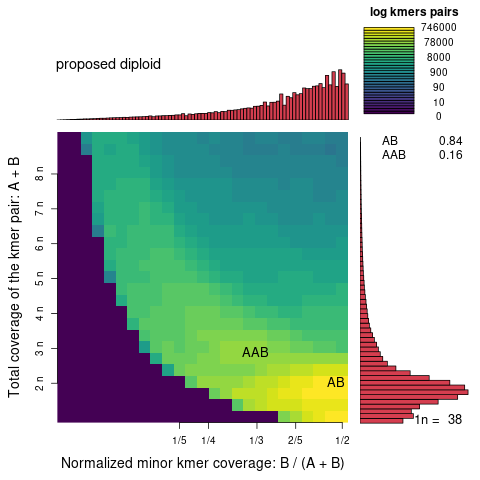

Supplement: Supplementary file 2 — Appendix S2. Smudgeplots for 32 Pinguicula species, each showing the proposed ploidy level and the k‐mer pair coverage used to estimate genome characteristics. [file AJB2-113-e70156-s005.zip › AppendixS2/P01_1.Pinguicula_acuminata.smudgeplot_smudgeplot_log10.png]

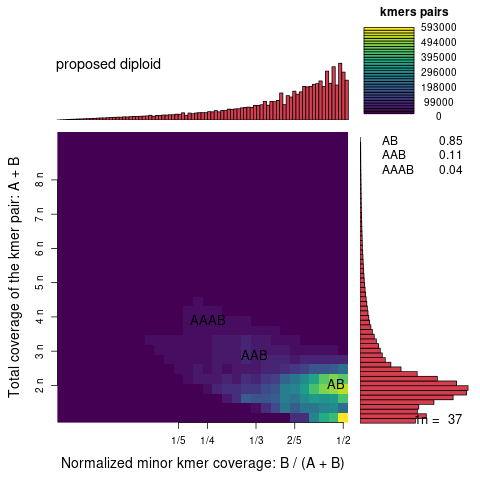

Supplement: Supplementary file 2 — Appendix S2. Smudgeplots for 32 Pinguicula species, each showing the proposed ploidy level and the k‐mer pair coverage used to estimate genome characteristics. [file AJB2-113-e70156-s005.zip › AppendixS2/P22.Pinguicula_macrophylla.smudgeplot_smudgeplot.png]

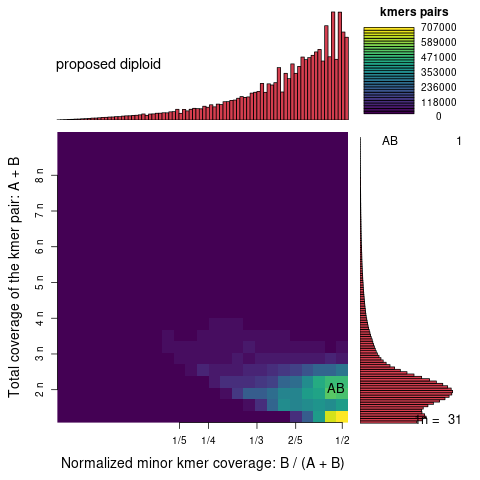

Supplement: Supplementary file 2 — Appendix S2. Smudgeplots for 32 Pinguicula species, each showing the proposed ploidy level and the k‐mer pair coverage used to estimate genome characteristics. [file AJB2-113-e70156-s005.zip › AppendixS2/P19.Pinguicula_kondoi.smudgeplot_smudgeplot.png]

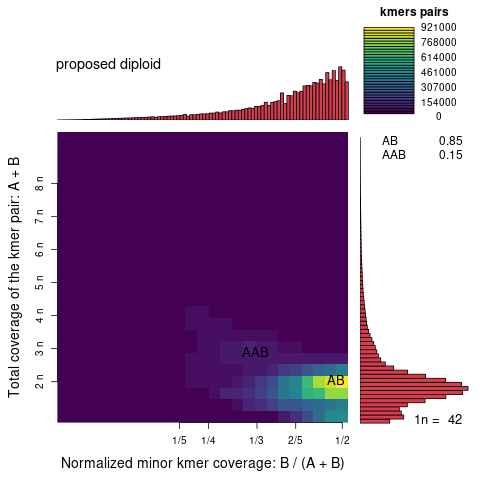

Supplement: Supplementary file 2 — Appendix S2. Smudgeplots for 32 Pinguicula species, each showing the proposed ploidy level and the k‐mer pair coverage used to estimate genome characteristics. [file AJB2-113-e70156-s005.zip › AppendixS2/P13_1.Pinguicula_gigantea.smudgeplot_smudgeplot.png]

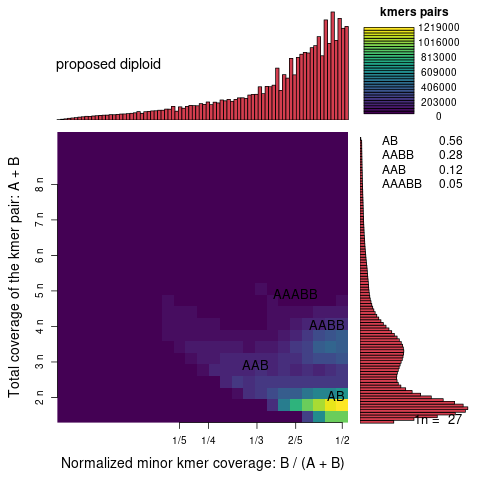

Supplement: Supplementary file 2 — Appendix S2. Smudgeplots for 32 Pinguicula species, each showing the proposed ploidy level and the k‐mer pair coverage used to estimate genome characteristics. [file AJB2-113-e70156-s005.zip › AppendixS2/P14.Pinguicula_gypsicola.smudgeplot_smudgeplot.png]

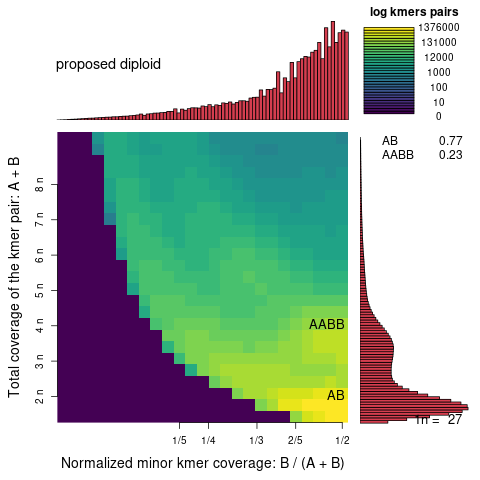

Supplement: Supplementary file 2 — Appendix S2. Smudgeplots for 32 Pinguicula species, each showing the proposed ploidy level and the k‐mer pair coverage used to estimate genome characteristics. [file AJB2-113-e70156-s005.zip › AppendixS2/P09.Pinguicula_debbertiana.smudgeplot_smudgeplot_log10.png]

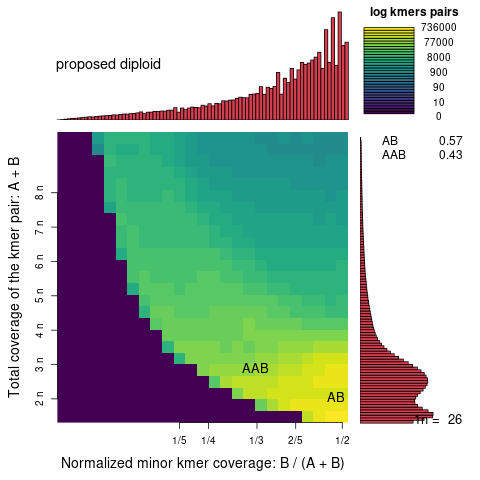

Supplement: Supplementary file 2 — Appendix S2. Smudgeplots for 32 Pinguicula species, each showing the proposed ploidy level and the k‐mer pair coverage used to estimate genome characteristics. [file AJB2-113-e70156-s005.zip › AppendixS2/P29_1.Pinguicula_oblongiloba.smudgeplot_smudgeplot_log10.png]

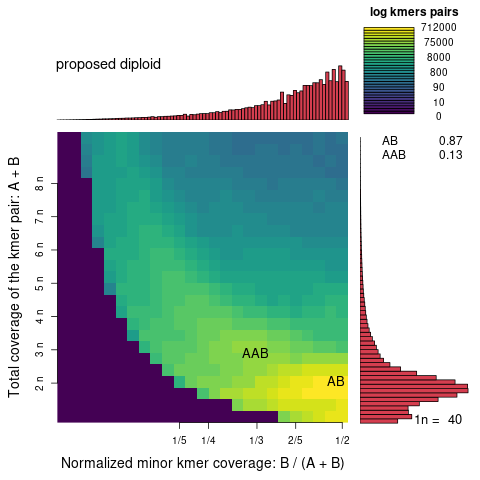

Supplement: Supplementary file 2 — Appendix S2. Smudgeplots for 32 Pinguicula species, each showing the proposed ploidy level and the k‐mer pair coverage used to estimate genome characteristics. [file AJB2-113-e70156-s005.zip › AppendixS2/P18.Pinguicula_immaculata.smudgeplot_smudgeplot_log10.png]

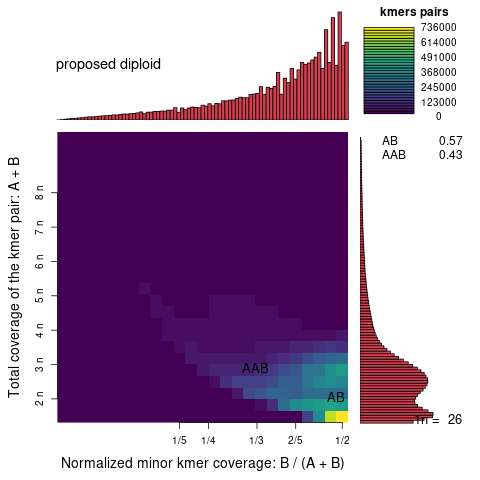

Supplement: Supplementary file 2 — Appendix S2. Smudgeplots for 32 Pinguicula species, each showing the proposed ploidy level and the k‐mer pair coverage used to estimate genome characteristics. [file AJB2-113-e70156-s005.zip › AppendixS2/P29_1.Pinguicula_oblongiloba.smudgeplot_smudgeplot.png]

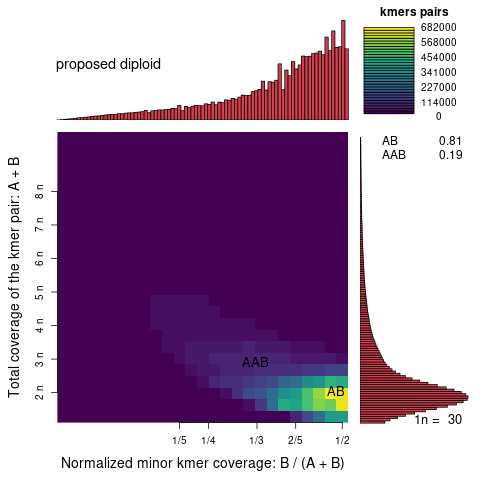

Supplement: Supplementary file 2 — Appendix S2. Smudgeplots for 32 Pinguicula species, each showing the proposed ploidy level and the k‐mer pair coverage used to estimate genome characteristics. [file AJB2-113-e70156-s005.zip › AppendixS2/P33_1.Pinguicula_robertiana.smudgeplot_smudgeplot.png]

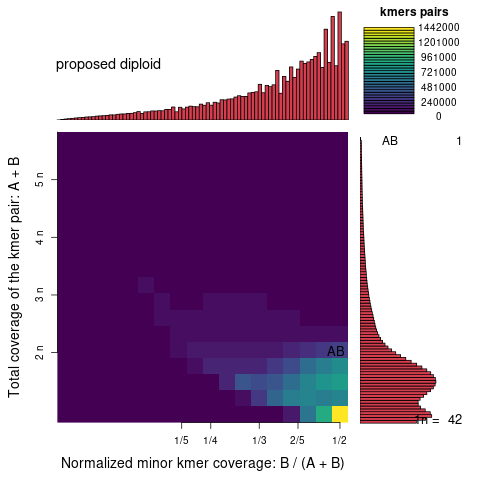

Supplement: Supplementary file 2 — Appendix S2. Smudgeplots for 32 Pinguicula species, each showing the proposed ploidy level and the k‐mer pair coverage used to estimate genome characteristics. [file AJB2-113-e70156-s005.zip › AppendixS2/P23.Pinguicula_martinezii.smudgeplot_smudgeplot.png]

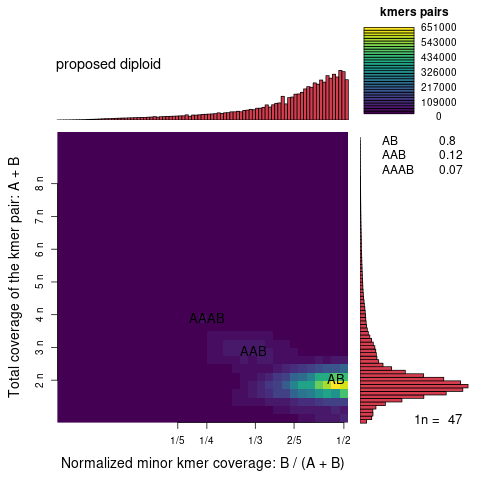

Supplement: Supplementary file 2 — Appendix S2. Smudgeplots for 32 Pinguicula species, each showing the proposed ploidy level and the k‐mer pair coverage used to estimate genome characteristics. [file AJB2-113-e70156-s005.zip › AppendixS2/P26_1.Pinguicula_moctezumae.smudgeplot_smudgeplot.png]

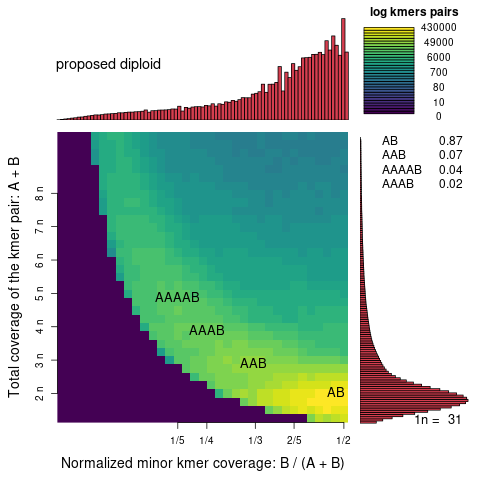

Supplement: Supplementary file 2 — Appendix S2. Smudgeplots for 32 Pinguicula species, each showing the proposed ploidy level and the k‐mer pair coverage used to estimate genome characteristics. [file AJB2-113-e70156-s005.zip › AppendixS2/P30_1.Pinguicula_orchidioides.smudgeplot_smudgeplot_log10.png]

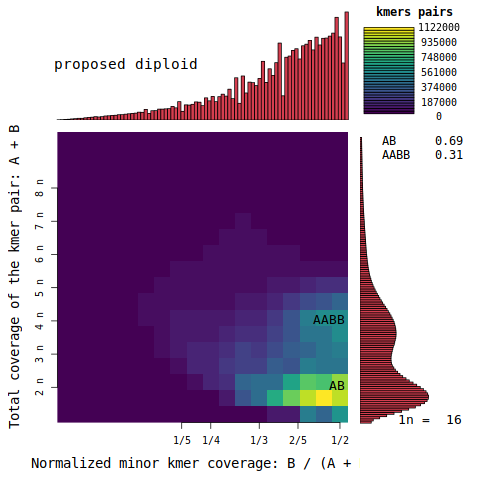

Supplement: Supplementary file 2 — Appendix S2. Smudgeplots for 32 Pinguicula species, each showing the proposed ploidy level and the k‐mer pair coverage used to estimate genome characteristics. [file AJB2-113-e70156-s005.zip › AppendixS2/P02.Pinguicula_agnata.smudgeplot_smudgeplot.png]

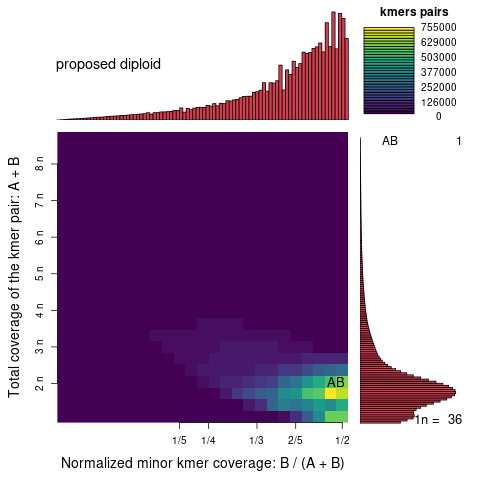

Supplement: Supplementary file 2 — Appendix S2. Smudgeplots for 32 Pinguicula species, each showing the proposed ploidy level and the k‐mer pair coverage used to estimate genome characteristics. [file AJB2-113-e70156-s005.zip › AppendixS2/P11_1.Pinguicula_elizabethiae.smudgeplot_smudgeplot.png]

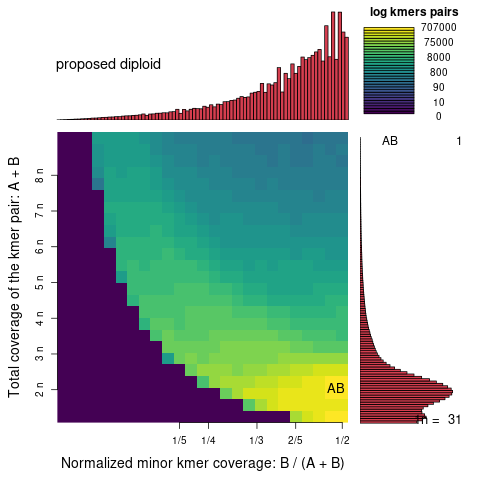

Supplement: Supplementary file 2 — Appendix S2. Smudgeplots for 32 Pinguicula species, each showing the proposed ploidy level and the k‐mer pair coverage used to estimate genome characteristics. [file AJB2-113-e70156-s005.zip › AppendixS2/P19.Pinguicula_kondoi.smudgeplot_smudgeplot_log10.png]

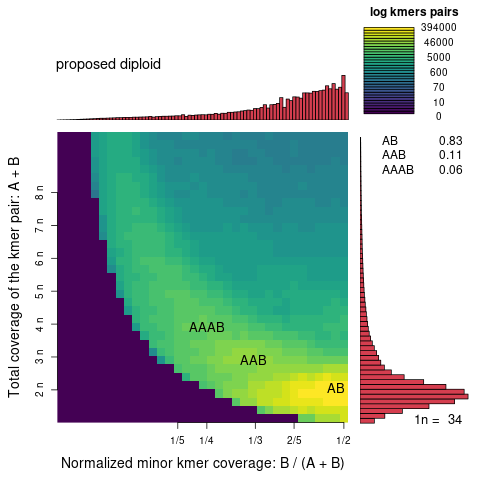

Supplement: Supplementary file 2 — Appendix S2. Smudgeplots for 32 Pinguicula species, each showing the proposed ploidy level and the k‐mer pair coverage used to estimate genome characteristics. [file AJB2-113-e70156-s005.zip › AppendixS2/P36_1.Pinguicula_zamudioana.smudgeplot_smudgeplot_log10.png]

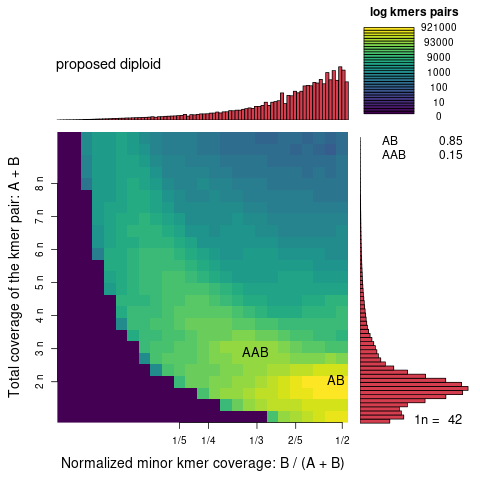

Supplement: Supplementary file 2 — Appendix S2. Smudgeplots for 32 Pinguicula species, each showing the proposed ploidy level and the k‐mer pair coverage used to estimate genome characteristics. [file AJB2-113-e70156-s005.zip › AppendixS2/P13_1.Pinguicula_gigantea.smudgeplot_smudgeplot_log10.png]
